# Flexible and self-powered temperature–pressure dual-parameter sensors using microstructure-frame-supported organic thermoelectric materials

**DOI:** 10.1038/ncomms9356

**Published:** 2015-09-21

**Authors:** Fengjiao Zhang, Yaping Zang, Dazhen Huang, Chong-an Di, Daoben Zhu

**Affiliations:** 1 Beijing National Laboratory for Molecular Sciences, Key Laboratory of Organic Solids, Institute of Chemistry, CAS, Beijing 100190, China; 2 University of Chinese Academy of Sciences, Beijing 100049, China

## Abstract

Skin-like temperature- and pressure-sensing capabilities are essential features for the next generation of artificial intelligent products. Previous studies of e-skin and smart elements have focused on flexible pressure sensors, whereas the simultaneous and sensitive detection of temperature and pressure with a single device remains a challenge. Here we report developing flexible dual-parameter temperature–pressure sensors based on microstructure-frame-supported organic thermoelectric (MFSOTE) materials. The effective transduction of temperature and pressure stimuli into two independent electrical signals permits the instantaneous sensing of temperature and pressure with an accurate temperature resolution of <0.1 K and a high-pressure-sensing sensitivity of up to 28.9 kPa^−1^. More importantly, these dual-parameter sensors can be self-powered with outstanding sensing performance. The excellent sensing properties of MFSOTE-based devices, together with their unique advantages of low cost and large-area fabrication, make MFSOTE materials possess promising applications in e-skin and health-monitoring elements.

## Abstract (web summary)

The construction of electronic skin requires simultaneous temperature and pressure detection. Here Zhang *et al.* utilize independent thermoelectric and piezoresistive effect in a single self-powered device, which shows a temperature resolution of <0.1 K and a pressure sensitivity of 28.9 kPa^−1^.

## Graphical abstract

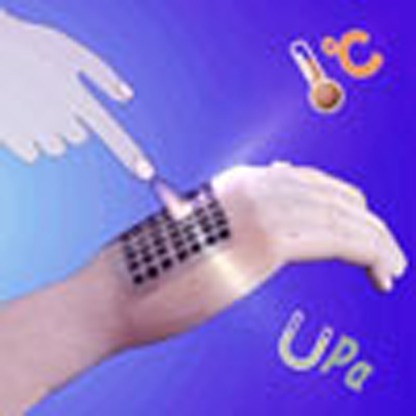

Flexible, low-cost temperature- and pressure-sensing devices are of profound importance in satisfying the urgent requirements for many advanced artificial intelligence applications^1^,^2^,^3^,^4^,^5^,^6^,^7^,^8^,^9^,^10^,^11^,^12^,^13^,^14^,^15^,^16^,^17^,^18^,^19^,^20^,^21^,^22^,^23^,^24^,^25^. Organic electronic devices, distinguished by their intrinsically low-cost and flexibility, are believed to be promising candidates for integrated smart systems. Recently, the first steps have been taken toward novel electronic skin and wearable elements based on organic materials, as reflected by the successful demonstration of many sensitive pressure sensors for healthcare applications^6^,^7^,^11^,^26^,^27^,^28^,^29^,^30^. The achievement of even more fascinating applications of organic electronics relies on the simultaneous sensing of pressure and temperature stimuli to mimic the functionality of human skin. The realization of this functionality, however, requires the transduction of multiple stimuli into a coupled or separated signal by the sensing elements. To date, only limited examples of temperature–pressure-sensing devices based on organic material have been demonstrated because of the critical requirements to the multifunctionality of the materials and unique geometry of the devices^31^,^32^,^33^,^34^.

The integration of individual pressure and temperature sensors into one pixel represents an efficient approach for achieving dual-parameter-sensing functionality^31^,^35^. Benefiting from the ingenious integration of organic devices, Someya *et al.*^31^ demonstrated conformable, flexible and large-area networks of pressure and temperature sensors with organic field-effect transistor (OFET) active matrixes. Despite this pioneering work, widespread application of these sensing elements has been limited by the sophisticated fabrication processes involved in fabricating three types of functional devices. An alternative approach is the development of bimodal sensors that transduce different input stimuli into a coupled signal within a single device^32^,^33^,^34^. Taking advantage of the integration of piezo-pyroelectric and piezo-thermoresistive materials in organic transistors, simultaneous detection of pressure and temperature has been achieved in an OFET via an exquisite combination of an AC gate bias operation and a current-decoupling analysis^34^. However, highly sensitive detection of different stimuli has not been realized, which is due in part to the multiple-signal interference in the decoupling analysis. These issues make construction of flexible, low-cost, sensitive organic dual-parameter sensors a great challenge.

The development of novel dual-parameter sensors that transduce different stimuli into separated signals can intrinsically minimize signal interference, thus allowing for the sensitive detection of both temperature and pressure in a single device without decoupling analysis. For most organic active devices with external power supplies, the current is usually an exclusive read-out signal, which impedes their use in dual-parameter sensing application. In comparison, many power generation devices, for example, organic solar cells and organic thermoelectric device^36^,^37^, can serve principally as ideal dual-parameter sensors because their output voltage is an additional read-out signal. More importantly, the power generation properties of these devices can facilitate the construction of flexible sensors with ultralow power consumption. Typical organic power generation devices, however, are not considered to be promising candidates for temperature–pressure-sensing applications because they are not quantitatively sensitive to these stimuli simultaneously.

Here we demonstrate temperature–pressure dual-parameter sensors utilizing microstructure-frame-supported organic thermoelectric (MFSOTE) materials. By taking advantage of independent thermoelectric and piezoresistive effects in a single MFSOTE device, simultaneous monitoring of temperature and pressure is realized by transducing external stimuli into separate electrical signals. Notably, the devices can be self-powered by a natural temperature gradient with a high-temperature detection resolution and pressure-sensing sensitivity of up to 0.1 K and 28.9 kPa^−1^, respectively. The excellent sensing performance, prominent flexibility and self-powered features of MFSOTE devices enable their promising applications in artificial intelligence and healthcare systems.

## Results

### Basic mechanism and device fabrication

**Figure 1:**
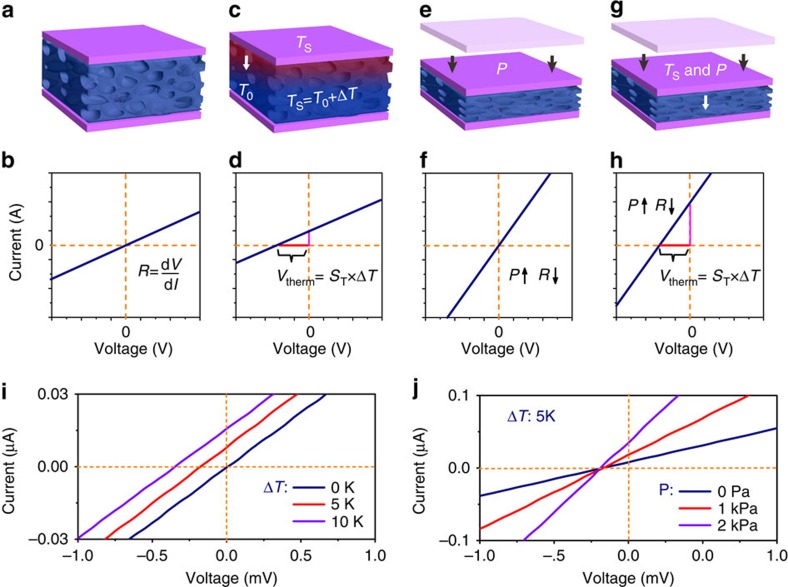
Illustrative schematic and electrical properties of MFSOTE devices. (**a**–**h**) Schematic illustration of temperature–pressure (*T*–*P*) sensing mechanism: (**a**,**b**) pristine. (**c**,**d**) a temperature gradient (Δ*T*) is applied across a MFSOTE device. (**e**,**f**) a pressure is loaded. (**g**,**h**) loading of a coupled temperature and pressure stimuli. Graph (**i**,**j**) show the measured *I*–*V* curves of a MFSOTE device taken at various Δ*T* (**i**) and different loading pressure (**j**). The time delay (**i**,**j**) between the contact and the measurement of the electrical signal is 10 s.

For a MFSOTE device, the active layer is constructed by the deposition of organic thermoelectric materials on deformable microstructure frames to enable their temperature and pressure-sensing properties. According to a typical thermoelectric mechanism, the generated voltage (*V*_therm_) of a MFSOTE is defined as *V*_therm_=*S*_T_ × Δ*T*, where *S*_T_ is the Seebeck coefficient and Δ*T* is the temperature gradient on the device. When the device is exposed to an object with coupled temperature and pressure stimuli, the temperature difference between the object and the device is detected via the thermoelectric effect, leading to instantaneous sensing of the surface temperature once the device possesses a skin-like constant base temperature (Fig. 1a–d). Meanwhile, the microstructure frame deforms as a result of an existing force, which results in a change in the resistance of the active layer as a function of the biased pressure (Fig. 1e–f). Therefore, temperature and pressure stimuli can be detected separately and simultaneously (Fig. 1g–h).

As mentioned above, the temperature- and pressure-sensing performance of a MFSOTE device is governed by the thermoelectric and pressure deformation properties of the MFSOTE materials. Using thermoelectric material and microstructured frame, namely, poly(3,4-ethylenedioxythiophene): poly(styrenesulfonate) (PEDOT:PSS) and porous Polyurethane (PU), we fabricated MFSOTE materials via the simple immersion of a microstructured PU (60 pores per inch (ppi)) in PEDOT:PSS solution with 5 vol% ethylene glycol (EG; Supplementary Fig. 1). The uniform PEDOT:PSS film was then deposited onto the PU networks (Supplementary Fig. 2). The device fabrication was completed with lamination of two conducting films on the active layer to achieve a sandwich device structure. Note that commercial materials were utilized as the functional materials, and the entire device fabrication process is easily scalable. All the results presented in the main manuscript have been obtained with the PU–PEDOT:PSS composite, while other conducting polymers have been tested and the results are presented in the Supplementary Information.

### Sensing characteristics of MFSOTE device

As shown in Fig. 1i, a typical *I*–*V* curve was observed when no temperature or pressure was applied, indicating the well-defined electrical properties of the MFSOTE devices. However, the *I*–*V* curve clearly shifted, with *V*_therm_ values of 173 and 345 μV under constant temperature differences of 5 and 10 K, respectively, because of the thermoelectric effect of the PEDOT:PSS layer. By comparison, the resistance of the MFSOTE device decreased from 8.8 to 5 kΩ under biased pressure ranging from 1 to 2 kPa (Fig. 1j, Supplementary Fig. 3). It should be mentioned that the temperature stimuli showed a limited effect on the electrical resistance, and the pressure signal exhibited a negligible effect on *V*_therm_ (Supplementary Fig. 4). These properties enabled separate temperature and pressure stimuli sensing using voltage and current change, respectively, as read-out signals.

**Figure 2:**
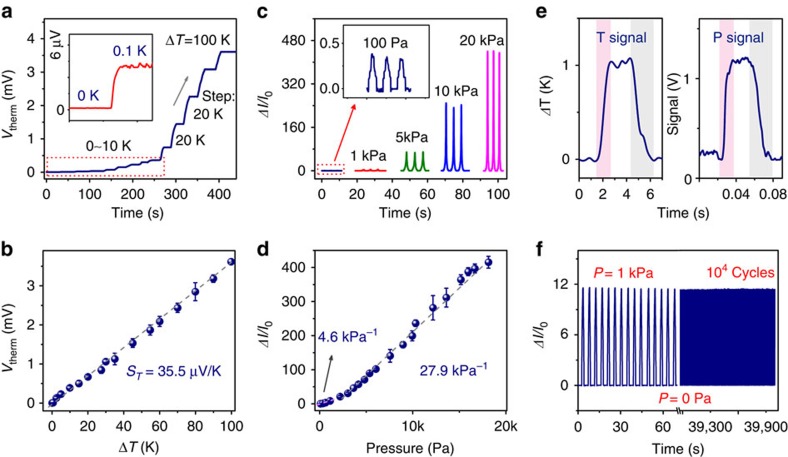
Electrical characterization of MFSOTE devices. (**a**) Output voltage of a MFSOTE device to a biased temperature gradient range of 0−100 K. The insert shows the magnified response signal of a MFSOTE device to a temperature gradient of 0.1 K. (**b**) Measured output voltage as a function of temperature gradient. (**c**) Current response of a MFSOTE device to various pressures at a constant voltage of 0.1 V. The insert shows the magnified response signal to the pressure of 100 Pa. (**d**) Current responses of the MFSOTE devices to various pressures. The error bars in graph (**b**,**d**) represent the s.d. (**e**) Time-resolved responses of a MFSOTE device to temperature and pressure stimuli. The pink and the grey zones represent the response and relaxation time, respectively. (**f**) The durability test of a MFSOTE device under a pressure of 1 kPa.

We first measured the temperature-gradient-dependent (Δ*T*) thermoelectric voltage generation of MFSOTE devices to assess the temperature-sensing response of the fabricated devices. Figure 2a,b present the measured output voltage as a function of the temperature difference ranging from 0.1 to 100 K. Notably, even a small Δ*T* of 0.1 K was clearly detected with a *V*_therm_ of >3 μV. It implies that an accurate temperature resolution of <0.1 K can be achieved when a typical microvotmeter (with internal resistance of 1 MΩ) is utilized to measure the *V*_therm_ signal (Supplementary Note 1). As a result of the well-defined linear *V*_therm_-Δ*T* relationship, a moderate *S*_T_ of 32.8±2.7 μV/K was extracted, which is consistent with the value of EG treated PEDOT:PSS film (31.1±3.1 μV/K). The reproducible and stable thermoelectric conversion enables sensitive detection of the surface temperature of the MFSOTE (Supplementary Figs 5 and 6).

Considering that the temperature of the bottom electrode (*T*_0_) might be heated up when the top electrode is in contact with a temperature signal (*T*_S_) for a long time, we deposited a Copper thermocouple on the bottom electrode and measured *T*_0_ when hot objects with different *T*_S_ are in contact with the device for different time (Supplementary Fig. 7). For a free-standing MFSOTE device, the temperature of the bottom electrode increased obviously once *T*_S_ increase from 25 to 75 °C. A different phenomenon was observed once the device is worn on the human body. When a hot object is in contact with the device for a short time, no obvious change of the *T*_0_ was observed owing to the constant temperature of the human skin. When the hot object was contact with the device for a long time, *T*_0_ increased once the *T*_S_ is higher than 60 °C. It should be noted that no obvious change in *T*_0_ was observed when the temperature of the hot electrode is lower than 60 °C. This result demonstrates that accurate temperature sensing can be achieved in a normal dynamic operation with a typical real-time environmental condition.

We also investigated the pressure-dependent performance of MFSOTEs to evaluate their pressure-sensing abilities. As shown in Fig. 2c,d, the devices exhibited markedly enhanced current with increasing pressure over a range from 0.1 to 20 kPa, even when operating at a low operating voltage of 0.1 V. The pressure sensitivity of our devices is defined as *S*=(Δ*I*/*I*_0_)/Δ*P*, where Δ*I* is the pressure-induced change in current, *I*_0_ is the initial current of the sensor without pressure loading and ΔP is the change in the applied pressure. The unique microstructure frame of our MFSOTE devices makes it possible to achieve a high-pressure-sensing sensitivity of 27.9 kPa^−1^. To our knowledge, this sensitivity is one of the most prominent values reported for a flexible resistive pressure sensor^13^,^20^. The outstanding temperature–pressure dual-parameter sensing performance of MFSOTE devices can satisfy the monitoring requirements for various applications.

For a MFSOTE device composed of PEDOT:PSS and microstructured PU, the temperature gradient-induced voltage generation is determined by the thermoelectric properties of PEDOT:PSS as mentioned above (*V*_therm_=*S*_T_ × Δ*T*). In the case of PEDOT:PSS (5 vol% EG), the environmental temperature possesses limited effect on the Seebeck coefficient in a temperature regime from 0 to 100 °C and biased pressure does not affect the Seebeck coefficient obviously (Supplementary Fig. 4). As a result, accurate temperature sensing can be realized.

The pressure-induced current change is determined by the contact resistance of electrode/MFOTE, contact resistance of neighbouring PU–PEDOT:PSS pores and resistance changes result from decreased thickness of the compressed devices (Supplementary Figs 8 and 9). All these resistance changes are dominated by the pressure-induced strain with two regimes, as reflected by the comparable piezoresistive responses of the device with mechanical responses of MFSOTE materials (Supplementary Figs 8–10). For our MFSOTE device based on PU–PEDOT:PSS, the strain–pressure relationship can be expressed by (a detailed discussion is provided in Supplementary Note 2):


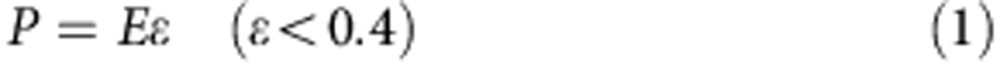



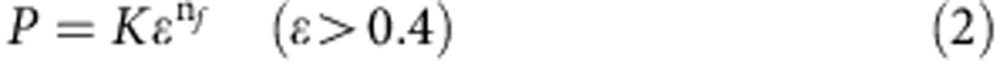


where *P* is the biased pressure, *E* is elastic modulus, *ɛ* is strain, *K* is a parameter related to the Young's modulus of the solid cell wall material, n_f_, which is affected by the density, is strain-hardening exponent of the foam obtained by fitting a power–law relation to the data points of the stress–strain curve. Given that elastic modulus and density are representative parameters that determined the stress–strain relationship of the MFSOTE in two different regime, the pressure-sensing performance of MFSOTE devices are dominated by the mechanical and structural properties of microstructure PU frames rather than PEDOT:PSS. This conclusion can be verified by the PU pore-density-dependent pressure-sensing performance of MFSOTE devices (Supplementary Figs 11 and 12). A larger pore density results in enhanced strain at a fixed pressure and leads to improved pressure-sensing performance. Notably, the pressure-sensing sensitivity is principally affected by the temperature-induced changes in the conductivity during the sensing process. However, in the case of PEDOT:PSS (5 vol% EG), the temperature has a negligible effect on the conductivity within a large temperature regime from 0 to 100 °C^38^. These unique properties make PEDOT:PSS (5 vol% EG) an excellent candidate for separate temperature–pressure dual-parameter sensors.

As the pressure and temperature detection of a MFSOTE device is determined by different materials with varied electrical parameters, we can draw a conclusion that the pressure- and temperature-sensing performance can be modulated by the supporting frames and thermoelectric materials independently. By depositing typical organic thermoelectric materials such as poly(3-hexylthiophene) (P3HT) and poly(2,5-bis(3-tetradecylthiophen-2-yl)thieno[3,2-b]thiophene) (PBTTT) onto porous PU supporting frames, we successfully constructed several MFSOTE devices with remarkable temperature–pressure-sensing performance (Supplementary Figs 13 and 14). A high Seebeck coefficient of up to 179−200 μV K^−1^, which facilitates sensitive temperature detection in a simple manner, was achieved. Although a slight decrease in pressure-sensing sensitivity (17.5−20.0 kPa^−1^) was observed because of solvent damage to the structure of the PU frames, this problem should be resolvable using alternate solvents. To confirm our deduction, we treated the PU frame with *o*-dichlorobenzene before the coating of the PEDOT:PSS. We obtained similar pressure-induced current changes with the PBTTT- and P3HT-based devices (Supplementary Fig. 15). In addition, different types of fabric materials including porous PU-based bandage, intertwined cellulose and grid-structured weaving cashmere can be used as the microstructured supporting frame to permit construction of different types of MFSOTE devices for various applications (Supplementary Fig. 16). Therefore, our proposed strategy of incorporating TE materials with a microstructure-supporting framework can serve as a general approach to the development of temperature–pressure sensors with tunable sensing performance.

### Response time and stability

The response time and stability are important parameters for physical sensors. Owing to their microstructure feature and thermoelectric sensing mechanism, our MFSOTE displayed an immediate response to an applied pressure and temperature signal. By utilizing an oscilloscope to measure the voltage change over a 10 kΩ resistor at a constant voltage of 2.2 V, the MFSOTE exhibited instantaneous response to an applied pressure of 1 kPa. The measured pressure response and relaxation time were <20 ms (Fig. 2e, Supplementary Fig. 17). In contrast to its rapid response to pressure, the device exhibited a moderate response time to temperature of <2 s for a temperature difference of 1 K (Fig. 2e). The response time is consistent with the thermal diffusion response time of the device (∼1.7 s), indicating that the temperature sensing response time is limited by the thermal diffusivity of the composite material (0.66 mm^2^ s^−1^). In addition, this temperature sensing response time, which is slightly affected by the temperature and pressure (Supplementary Figs 18 and 19), is consistent with those of many commercial and previously reported temperature sensors^39^,^40^. It is worth noting that both the sensitivity and response time of our fabricated devices can meet the requirements of many artificial intelligent systems. More importantly, the MFSOTE devices exhibit excellent stability, as demonstrated by their negligible environmental temperature-dependent Seebeck coefficient and resistance in a temperature regime of 0–100 °C (Supplementary Fig. 4c), their steady current response to a load pressure of 1 kPa, and their sustained thermoelectric performance after 10^4^ cycles in a loading–unloading test (Fig. 2f and Supplementary Fig. 20).

### Self-powered operation of the MFSOTE devices

**Figure 3:**
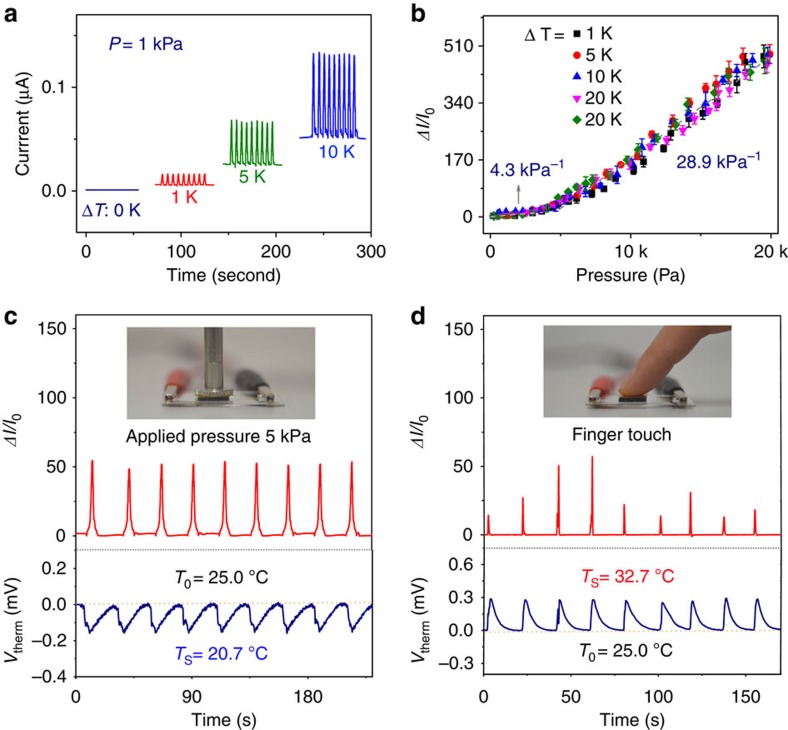
Self-powered sensing performance of MFSOTE devices. (**a**) Current response of a self-powered MFSOTE sensor to a pressure of 1 kPa taken under various temperature differences (0, 1, 5 and 10 K). (**b**) Pressure response of current changes driven by various temperature gradients. The error bars represent the s.d. (**c**,**d**) Plots showing the current and output voltage responses of a self-powered MFSOTE sensor to loading–unloading cycles of (**c**) a constant pressure of 5 kPa and (**d**) finger touch. The sensing temperature on the top surface is calculated by *T*_S_=*V*_therm_/*S*_T_+*T*_0_, where *T*_0_ is the ambient temperature (25.0 °C).

The construction of ultralow power consumption devices or self-powered sensing devices represents a significant step forward for the development of sustainable flexible sensors. For a MFSOTE device, surface temperature sensing can be accomplished without an additional power supply, because of the intrinsic thermoelectric temperature sensing mechanism. Although the PEDOT:PSS thermoelectric materials coated onto the surface of the microstructure-supporting frame can be utilized principally as a self-integrated power supply for the detection of the biased pressure, the output voltage is typically lower than 1 mV in a natural environment. Notably, we observed that the pressure-sensing performance of the MFSOTE is independent of the biased voltage over a wide range, from 30 μV to 1.5 V (Supplementary Fig. 21). The MFSOTE device can therefore serve as self-powered device (without consideration of the measurement circuits) once a small temperature gradient is available. As shown in Fig. 3a,b, comparable current responses to a fixed pressure were observed when the temperature gradient was maintained at 1−30 K (with a thermoelectric voltage of >30 μV). In particular, high self-powered pressure-sensing sensitivities of 4.3 and 28.9 kPa^−1^ were realized regardless of the temperature gradient in the low (<3 kPa) and high (>3 kPa) pressure regimes, respectively (Fig. 3b, Supplementary Fig. 22). These values are comparable to that of a device operating with an extra power supply. Since a temperature gradient of 1 K or more can be created easily in a natural environment, our devices can be self-powered for practical applications.

To evaluate the self-powered and simultaneous sensing abilities of the constructed devices, we monitored the current change and output voltage response of a MFSOTE device to temperature–pressure loading–unloading cycles (Fig. 3c,d). The MFSOTE device demonstrated a repeatable current change and *V*_therm_ response to a combined signal under a fixed pressure of 5 kPa and a temperature of 20.7 °C. The device recognized the slight difference in pressure among a series of gentle finger touches (1 to 5 kPa) on a single MFSOTE, whereas the monitored temperature (32.7 °C) remained unchanged because of the constant temperature of the finger. The self-powered and dual-parameter-sensing features of MFSOTE devices imply that the devices can potentially be used for long-term monitoring (Supplementary Movie 1). As a demonstration, we constructed an MFSOTE device on a PU bandage for self-powered monitoring of wrist pulses (Supplementary Fig. 23). Supplementary Fig. 23c presents real-time pressure wave monitoring under normal conditions and after physical exercise. A typical pulse pressure shape was obtained with distinguishable peaks^41^. The artery pulse frequency in a calm state is 75 beats per min, and the pulse beats faster with a frequency of ∼105 beats per min, during exercise.

**Figure 4:**
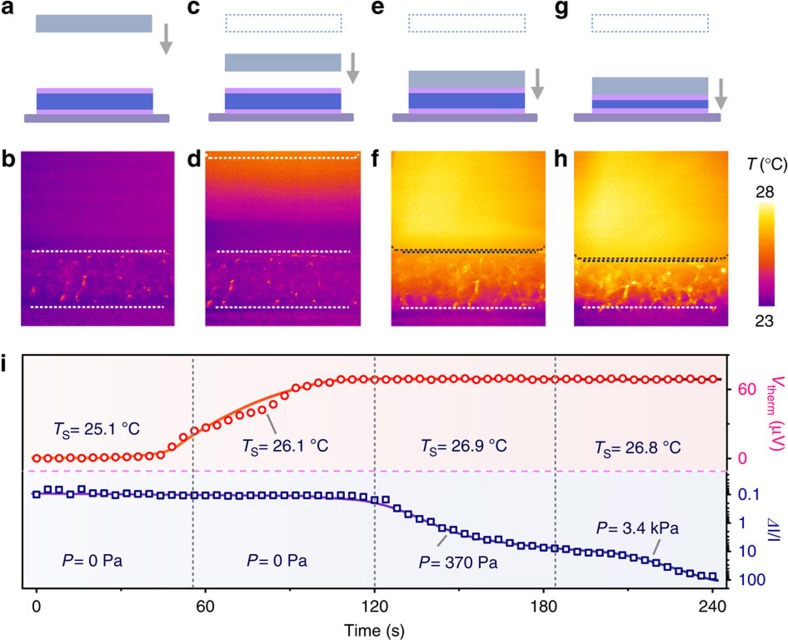
Real-time monitoring of temperature and pressure. (**a**,**c**,**e**,**g**) Schematic diagram and (**b**,**d**,**f**,**h**) infrared thermal images of a MFSOTE device approached by a Peltier element (27 °C). The temperature distribution was recorded simultaneously using a FLIR A300. The arrow indicates the moving direction of the Peltier element. (**i**) Plots of the real-time output voltage and current responses of a MFSOTE device approached by a Peltier element (27 °C).

Considering the thermal diffusion in air, contact-free sensing and monitoring of environmental temperature should be achieved when the base temperature of the MFSOTE is maintained. Figure 4 presents the result of a real-time monitoring test of a self-powered MFSOTE device when a heated element approaches our sensing device. In contrast to the negligible changes in both temperature and pressure when the heating device was >5 mm from the MFSOTE device, an obvious increase in temperature was detected after the distance decreased to <2 mm (Fig. 4a–d,i). When mechanical contact occurred, the surface temperature was maintained at a high value (26.8 °C), and the monitored pressure increased steadily from 100 Pa to 5 kPa with the movement of the step motor (Fig. 4e–i). The results were confirmed by the contact-free sensing of the thermal signal of an approaching finger and outdoor temperature with a wearable MFSOTE device (Supplementary Fig. 23d–f). This property enables contact-free thermal-stimuli mapping (Supplementary Fig. 24) and/or intelligent operation of novel smart devices.

### Self-integrated power generation properties of sensing array

**Figure 5:**
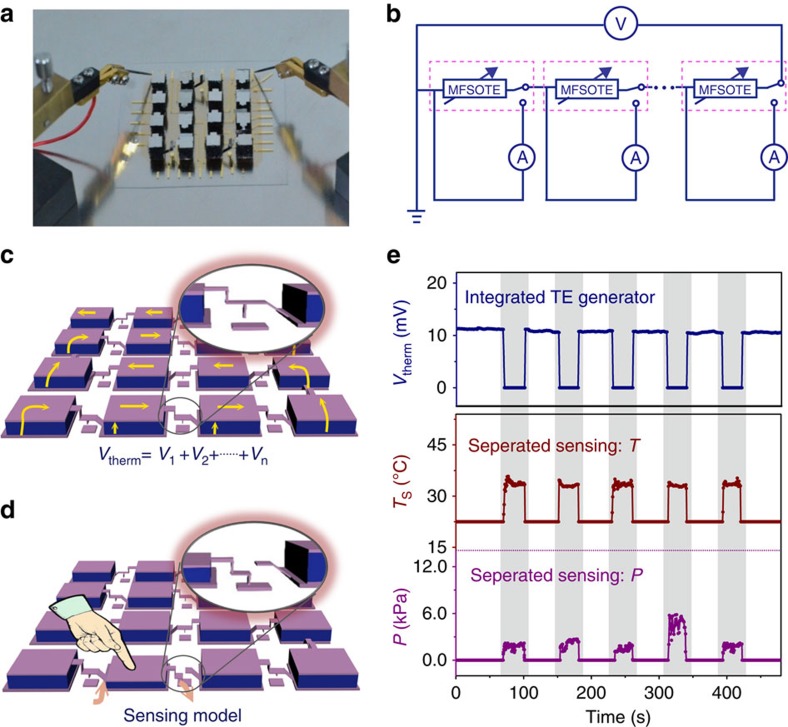
Multifunctional application of MFSOTE-based-sensing array. Graphs (**a**,**b**) show a photograph and circuit diagram, respectively, of an MFSOTE array with 4 × 4 pixels. Graphs (**c**,**d**) show schematic illustrations of the integrated array: (**c**) All the devices connected in series can be used as an electricity generator for harvesting energy. (**d**) The device switches to a self-powered dual-parameter sensor model when subjected to an external pressure. The insets in graph (**c**,**d**) show magnified views of the circuit connection between two pixels under unloading and loading conditions, respectively. (**e**) The thermoelectric voltage (top), temperature (middle), and pressure (bottom) responses of a MFSOTE array to five finger-touch cycles. The bottom surface of the MFSOTE array is heated with a Peltier element.

As an intrinsic thermoelectric device, our fabricated MFSOTE devices can also be utilized as electricity generators. Interestingly, the devices can be made to switch from power generation model to stimuli sensing model by the introduction of a pressure-induced switch between two MFSOTEs in a device array (a detailed description is provided in Methods section and Supplementary Figs 25 and 26). Figure 5a,b present a photograph and circuit diagram, respectively, of a MFSOTE array with 4 × 4 pixel devices. When a temperature gradient was applied to the device array, the devices operated in the power generation mode because the MFSOTE devices were connected in series to produce an accumulated output voltage (Fig. 5c). Once a pressure of ≥1 kPa is loaded on a device, the switch can disconnect the MFSOTE device from the other devices to allow for self-powered monitoring of pressure and temperature stimuli (Fig. 5d). As an example, we observed a high voltage of 10.1 mV when a moderate temperature difference of 18 K was loaded on the constructed device array (Fig. 5e). A gentle finger touch on a single device induced a reproducible switch from the power generation model to the sensing model, as indicated by the instantaneously detected temperature and pressure readings of 32.5 °C and 1−6 kPa, respectively, during five touch cycles. Moreover, effective mapping of sensing stimuli should be achievable since the separate signal can be recorded even more than one pixel is loaded simultaneously on the device array (Supplementary Figs 27 and 28). The combination of the power generation abilities of our MFSOTE devices, and their excellent sensing performance, makes the devices applicable as energy-harvesting-sensing elements.

### Flexible sensing array for artificial intelligent application

**Figure 6:**
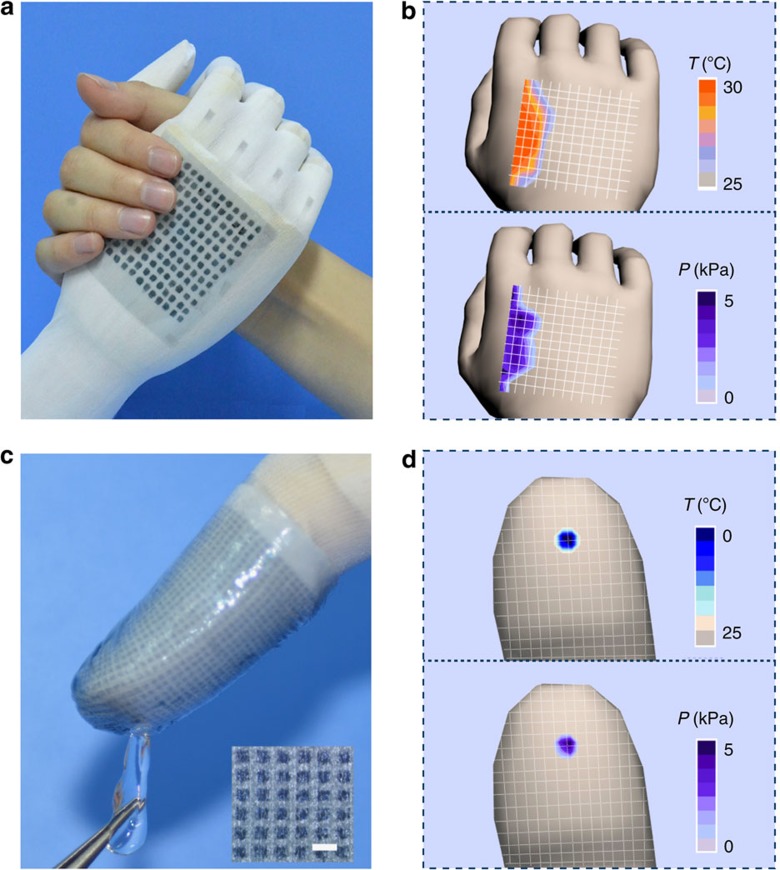
Photographs and performance of flexible MFSOTE matrixes. (**a**) Photograph of a prosthetic hand arm-wrestling with an adult woman. Graph (**b**) displays the temperature and pressure mapping profiles of pixel signals on the back of the prosthetic hand. Graph (**c**) shows an e-finger touching an ice cube. The inset shows the optical microscope image of the inkjet-printed MFSOTE matrix. Scale bar, 1 mm. (**d**) Photograph temperature and pressure mappings of the sensing array.

To satisfy the critical requirements of wearable intelligent systems and healthcare applications, the construction of a sensing MFSOTE array with remarkable flexibility is required to produce spatially resolved sensing products. Of particular note is that a simple integration method is highly desired to ensure low cost and widespread applicability. We therefore constructed a flexible 12 × 12 pixel MFSOTE array with dimensions of 4.6 × 4.6 cm^2^ on a semitransparent fabric glove (with a thickness of 500 μm) using a simple stamp-printing approach (Fig. 6a,b). All the devices displayed comparable sensing performance to a coupled temperature–pressure signal. By virtue of its uniform sensing ability and excellent flexibility, the MFSOTE array can be worn on a prosthetic hand to achieve spatially resolved images with subtle imaging features for both temperature and pressure. Figure 6a shows a photograph of a prosthetic hand arm-wrestling with an adult woman. The contact information was collected by monitoring the temperature and pressure on a reconstructed map, as illustrated in Fig. 6b. The changes in the temperature and biased pressure corresponded well to the colours of the pixels in the separated distribution map (Fig. 6). It is worth noting that the device can operate well on a high temperature of 130 °C (Supplementary Fig. 29), enabling their promising applications in robotics and intelligent wearable elements even to protect the hand to take hot object.

As one of the most susceptible part of human skin, fingertip can discriminate temperature–pressure stimuli with particularly high spatial resolution. It makes construction of artificial fingertip a more challenging task than that of a typical e-skin. Therefore, the construction of highly integrated MFSOTE array is of vital importance to meet the requirements of so-called e-finger. By utilizing inkjet-printing technique, we fabricated a MFSOTE array of 2 × 3 cm^2^ area (0.25 mm^2^ for each pixel) with 1,350 pixels on a fabric frame. Figure 6c shows photographs of an inkjet-printed MFSOTE matrix on a fingertip. When the fingertip touches a tiny ice cube with a contact area of 1.4 × 1.4 mm^2^ (4 pixels), spatially resolved pressure (2−3 kPa) and temperature (0−5 °C) information was collected on a reconstructed map (Fig. 6d). These results imply an excellent spatial resolution and dual-parameter-sensing capability of our sensing array.

## Discussion

MFSOTE not only allow microstructure-frame materials possess excellent electrical properties, but also endow organic thermoelectric materials with pressure-sensing features. The unique properties of the MFSOTE enable a dual-parameter sensing mechanism, wherein temperature–pressure coupled stimuli are quantitatively transduced into separated voltage and current change signals via the thermoelectric and piezoresistive effects, respectively. This capability makes MFSOTEs promising active materials for temperature–pressure-sensing elements. We have demonstrated that several microstructured materials (porous PU, intertwined cellulose and so on) and organic thermoelectric material (PEDOT:PSS, P3HT and PBTTT) can be utilized to fabricate flexible MFSOTE devices, thereby indicating that the strategy is generally applicable to various functional materials. Given that the functional materials are commercially available and our device fabrication processes are compatible with solution processing techniques, the development of large-area and low-cost MFSOTE devices are expected.

In addition to simultaneous dual-parameter sensing functionality, the MFSOTE devices exhibited an unexpected self-powered pressure-sensing sensitivity of >20 kPa^−1^ and an accurate temperature resolution of <0.1 K, when porous PU and PEDOT:PSS (5 vol% EG) serve as the supporting frame and thermoelectric material, respectively. The device performance is among the state-of-the-art performances of organic pressure–temperature sensors^31^,^34^. The prominent self-powered performance of the fabricated devices, together with their rapid response time and good stability, suggest that MFSOTEs are promising candidates for many applications. For example, benefitting from the temperature–pressure-sensing properties of MFSOTE, various sensing models, including pressure-, pressure/temperature- and temperature-sensing models, can be achieved in a single device in a form that fulfills the requirements of contact-induced and/or contact-free operation of novel intelligent systems. Moreover, the power-generating properties, outstanding flexibility and remarkable sensing properties of MFSOTE devices demonstrate their potential for long-term monitoring applications in wearable elements. Finally, MFSOTE devices can be inkjet-printed into a flexible array on fibres with high resolution that mimic the functionality of human fingertip. These features suggest that MFSOTEs are promising candidates with potential applications in skin-like intelligent devices. One limitation of the MFSOTE device is the possible temperature shift of the bottom electrode when a very hot object is in contact with the device for a long period of time, which impedes the accurate measurement of the top electrode via the thermoelectric mechanism. An important research direction of MFSOTE, therefore, might be the simple integration of temperature calibration elements in the MFSOTE array for more accurate mapping of the temperature for special smart applications.

On the basis of the sensing mechanism of the MFSOTE materials, the temperature and pressure-sensing performance is determined by the thermoelectric and mechanical properties of the composite. The temperature-sensing performance is dominated by the thermoelectric properties of the coated conducting polymers which should possess high Seebeck coefficient to enable efficient thermoelectrical signal conversion for accurate sensing of the temperature, good electrical conductivity to ensure low internal resistance for efficient measurement of the electrical signal, and negligible changes in Seebeck coefficient and electrical conductivity upon environmental temperature for real-life applications. Considering the fact that a high Seebeck coefficient and high conductivity are favourable for promising thermoelectric candidates, an organic thermoelectric material with a high-power factor may be suitable for MFSOTE application. In comparison, the piezoresistive properties of MFSOTE are determined by the mechanical properties of the supporting frames, rather than the electrical properties of thermoelectric materials. The achievement of high-pressure-sensing performance relies on the utilization of microstructured frames with optimized pore density and elastic modulus to realize a maximum variation of the resistance upon pressure. Finally, a high thermal resistance of the composite is preferable to maintain a large and stable temperature gradient for a long period of time, which is necessary to meet the critical requirements of realistic temperature–pressure-sensing applications.

In conclusion, we demonstrate the concept for constructing MFSOTE devices, that relies on the combination of organic thermoelectric materials and a microstructured supporting frame. This construction method is a simple but powerful strategy for the development of multifunctional sensors. The incorporation of piezoresistive and thermoelectric mechanisms enables the simultaneous detection of temperature and pressure stimuli without an additional decoupling process, and even features a self-powered pressure sensitivity and temperature-sensing accuracy of >20 kPa^−1^ and <0.1 K, respectively. More importantly, as evidenced by the prominent temperature–pressure stimulus mapping ability of a wearable integrated array, the MFSOTE devices hold promise for use as intelligent elements in a wide range of robotics and health-monitoring products.

## Methods

### MFSOTE-based device fabrication

The microstructured polyurethane (PU) frames (60 pores per inch (ppi), with the thickness of 1.8 mm) were washed with deionized water and blown dry using a nitrogen gun. They were then immersed in PEDOT:PSS (Clevios PH1000 from Heroeus; containing 5 vol% EG) and dried at 100 °C for 10 min. As a result, the PU frames were uniformly coated with the PEDOT:PSS film (0.4 μl mm^−3^), thus generating the MFSOTE material. The prepared materials were assembled into dual-parameter sensors by sandwiching MFSOTE material between two gold-modified aluminum electrodes.

In addition, different types of supporting frames including porous polyurethane (PU)-based bandage, intertwined cellulose and grid-structured weaving cashmere, as well as several organic thermoelectric materials such as P3HT and PBTTT were utilized to fabricate MFSOTE-based-sensing devices via a similar processing technique.

### MFSOTE-based array

For the fabrication of the MFSOTE-based self-integrated power generators, a glass substrate was sequentially cleaned with deionized water, alcohol and acetone. Patterned Au electrodes were evaporated onto the substrate using a shadow mask. PU-based MFSOTE materials (3 × 3 mm^2^, with a thickness of 1.8 mm) were prepared as previously described. Thereafter, the top and bottom electrode, which were consisted of two metal sheets with fixed shape, were utilized to create switchable circuit (described in Supplementary Fig. 25) for generator/sensor dual-model application.

A wearable sensing matrix with remarkable flexibility (Fig. 6a) was constructed using a simple stamp-printing method. A semitransparent fabric (with a thickness of 500 μm) was washed with deionized water and used as the microstructured frame. The patterned PEDOT:PSS pixels (12 × 12 pixels, the area of each pixel is 1.8 × 1.8 mm^2^) were subsequently prepared using a stamp-printing method and dried at 100 °C. A polyethylene terephthalate (PET) film (25 μm in thickness) with a patterned Ti/Au array was assembled inside the fabric as the bottom electrode. Meanwhile, another PEDOT:PSS (5 vol% EG)-coated ITO/PET film was laminated and affixed to the MFSOTE pixels as the top electrode on the outside face of the fabric. The high-resolution MFSOTE matrix was fabricated by inkjet-printing (Microfab Jetlab-II system) PEDOT:PSS (5 vol% EG) on the semitransparent fabric. Thereafter, a PET film with patterned Ti/Au and an Au-coated PDMS (20 μm) were laminated on the device as electrodes.

### Device characterization

The electric properties were measured using an Agilent B2902 precision source/measure unit under ambient conditions. The resistance was measured using two-probe technique. For the temperature-sensing tests, a Peltier element was used to create a temperature gradient, and the temperature measurements were performed with an FLIR A300. The temperatures were calibrated by thermocouples (Supplementary Fig. 30). The morphologies of the MFSOTE materials were characterized using scanning electron microscopy (SEM, Hitachi S-4800).

The thermal diffusivity is measured using a laser flash technique (LFA 447 NanoFlash, Netzsch), while thermal resistance is measured using Heat flow meter apparatus. The thermal diffusivity of a typical MFSOTE device is 0.66 mm^2^ s^−1^, which is slightly higher than that of PU foam and comparable with that of PEDOT:PSS. The measured thermal resistance is 0.18 m^2^K W^−1^ (with a thickness of 5 mm to meet thermal resistance measurement requirements).

The pressure-sensing properties of the sensors were characterized by applying an external load using a force gauge (Mark-10 025-E) in combination with a highly configurable motorized stand (EMS301-CP). The area was calculated on the basis of the contact surface. An oscilloscope (Agilent DSO-X 3052 A) was used to record the signals by measuring the change in the voltage drop across a 10-kΩ resistor, which was connected to the MFSOTE sensor in series. The measurement and signal addressing of MFSOTE array with self-integrated power-generating properties was realized through the integration of matrix switch system with B2902 precision source/measure unit (Supplementary Fig. 27).

### Measurement of seebeck coefficient

For the measurement of Seebeck coefficient of the MFSOTE and PEDOT:PSS thin film, two Peltier elements were used to create a temperature gradient, and the temperature measurement were performed using a FLIR A300 calibrated by Agilent U1273A. The thickness of MFSOTE materials for measurement of Seebeck coefficient is 2 mm. To measure the Seebeck coefficient of PEDOT:PSS film, we constructed devices with channel length of 2 mm and narrow, parallel strip electrodes (electrode width and length of 60 μm and 5 mm, respectively) to avoid systematic errors induced by the device geometry^42^. The Seebeck coefficient of the PEDOT:PSS film used in our work is 31.1±3.1 μV K^−1^, which is consistent with that of the MFSOTE materials (32.8±2.7 μV K^−1^).

## Additional information

**How to cite this article:** Zhang, F. *et al.* Flexible and self-powered temperature–pressure dual-parameter sensors using microstructure-frame-supported organic thermoelectric materials. *Nat. Commun.* 6:8356 doi: 10.1038/ncomms9356 (2015).

## Supplementary Material

Supplementary Information Supplementary Figures 1-30, Supplementary Notes 1-2 and Supplementary References

Supplementary Movie 1 Self-powered and dual-parameter sensing features of MFSOTE devices.
