# Supplementary material for: Flexible and self-powered temperature–pressure dual-parameter sensors using microstructure-frame-supported organic thermoelectric materials
Source: Nat Commun. 2015 Sep 21;6:8356. doi: 10.1038/ncomms9356 (PMC4595753; doi:10.1038/ncomms9356)
Supplement: Supplementary Information — Supplementary Figures 1-30, Supplementary Notes 1-2 and Supplementary References [file ncomms9356-s1.pdf]

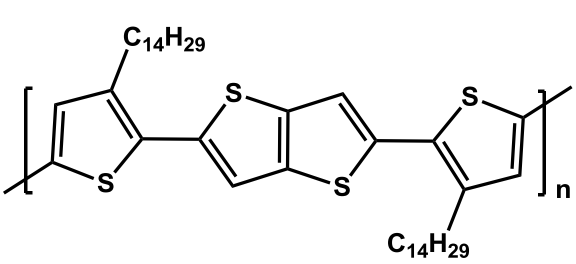

PBTTT

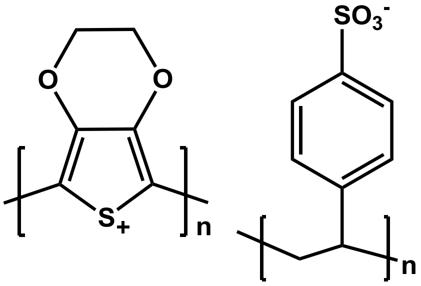

PEDOT:PSS

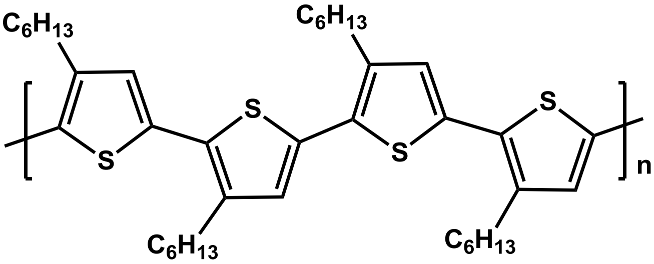

P3HT

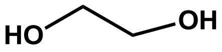

EG

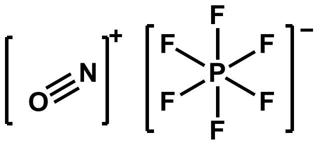

$NOPF_6$

Supplementary Figure 1 | Molecular structures involved in MFSOTE materials.

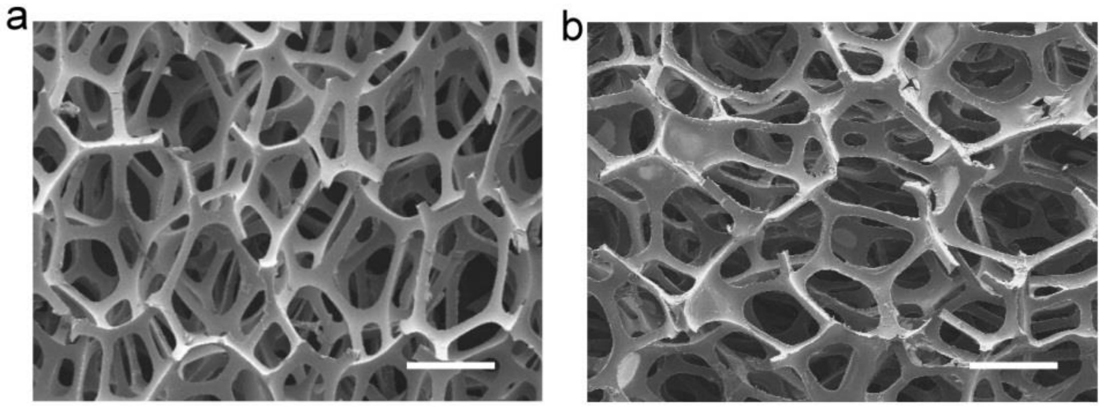

Supplementary Figure 2 | Microstructure characterization. SEM images of the microstructured PU without (**a**) and with (**b**) coated PEDOT:PSS. Scale bars: 500 μm.

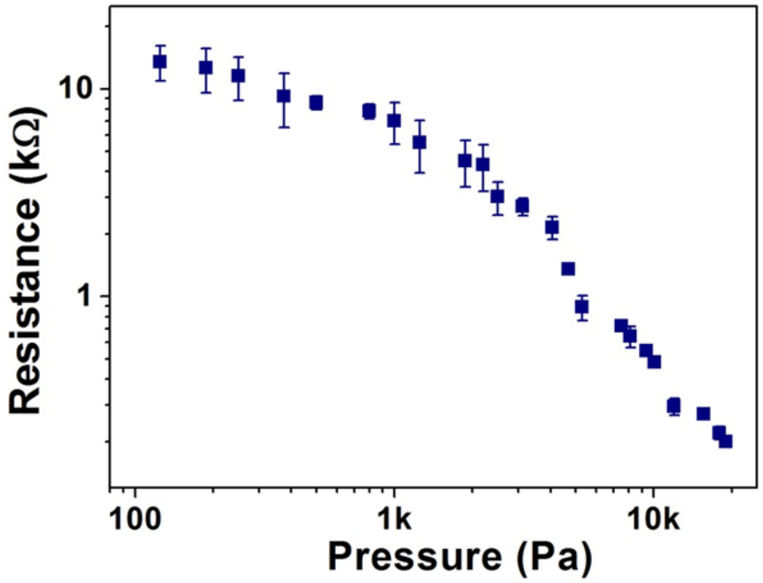

Supplementary Figure 3 | Pressure dependent resistance of MFSOTE devices. The measurements are taken at room temperature of 25.0 ℃. The error bars were calculated from more than five devices.

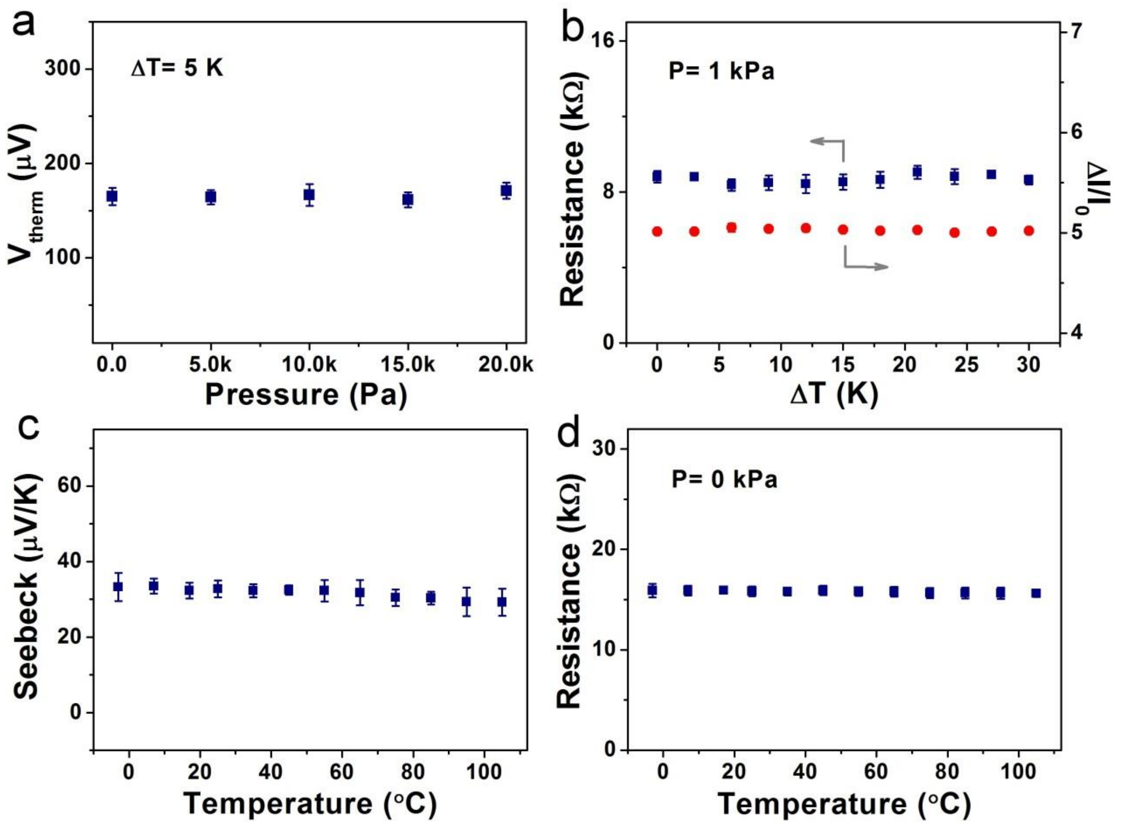

Supplementary Figure 4 | Separate pressure and temperature response of MFSOTE devices. (**a**) Measured thermal voltage as a function of pressure with a constant temperature difference of 5 K. (**b**) Electrical resistance and current change of MFSOTE devices as a function of temperature difference. The devices are measured under a pressure of 1 kPa. (**c**) Seebeck coefficient and (**d**) Resistance of the MFSOTE devices as a function of temperature with a pressure of 0 Pa. The time delay between the contact and the measurement of the electrical signal is 10 seconds. The error bars represent the standard deviation.

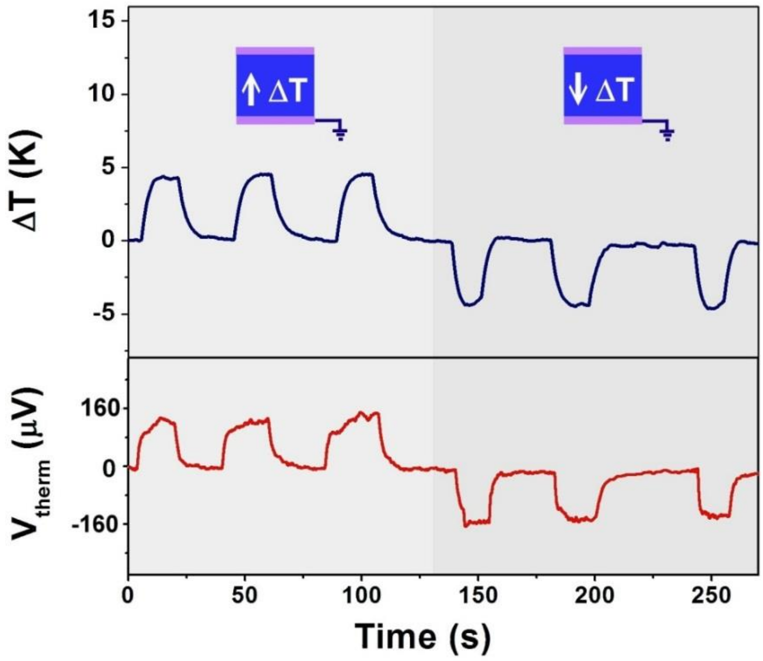

Supplementary Figure 5 | Temperature response of MFSOTE materials. Actual temperature gradients measured by IR sensor (top) and corresponding thermal voltage response of MFSOTE based devices (bottom).

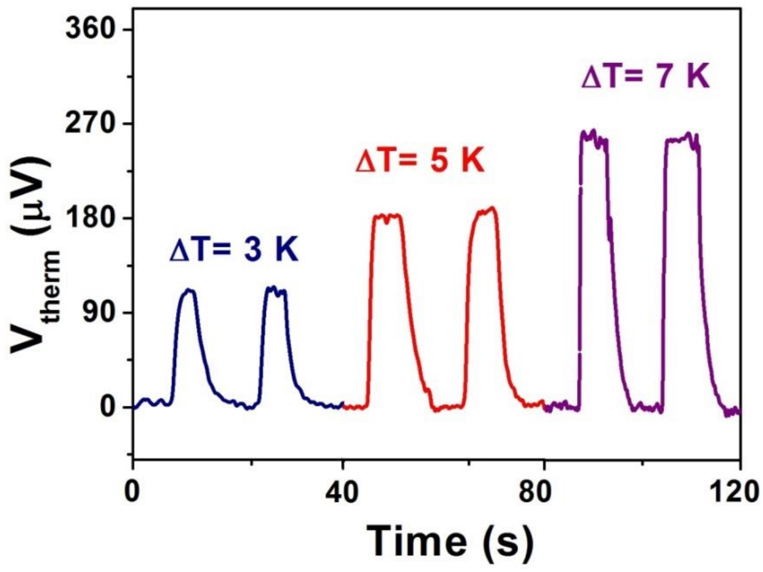

Supplementary Figure 6 | Temperature sensing test. The voltage response of a MFSOTE device to various temperature differences of 3 K, 5 K and 7 K, respectively.

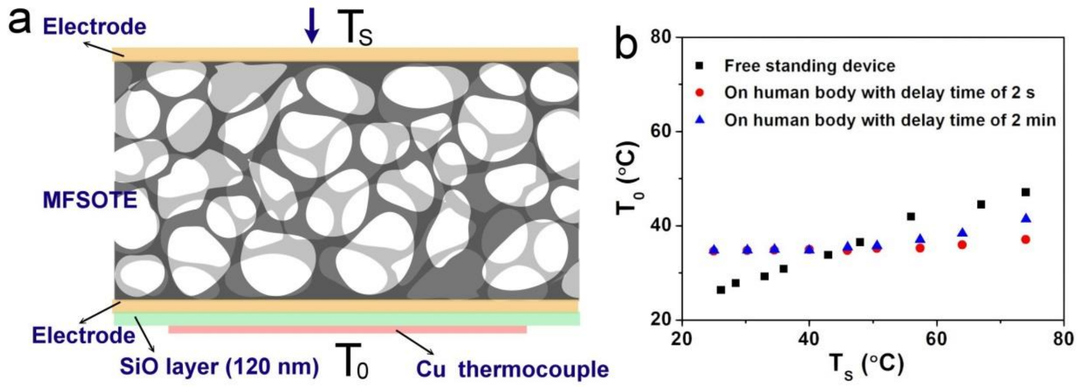

Supplementary Figure 7 | Temperature sensing stability. (**a**) Schematic illustration of a MFSOTE device integrated with a Cu thermocouple. (**b**) Temperature of the bottom electrode when a hot object is in contact with the top electrode for 2 minutes. The device is on the glass substrate and worn on human body.

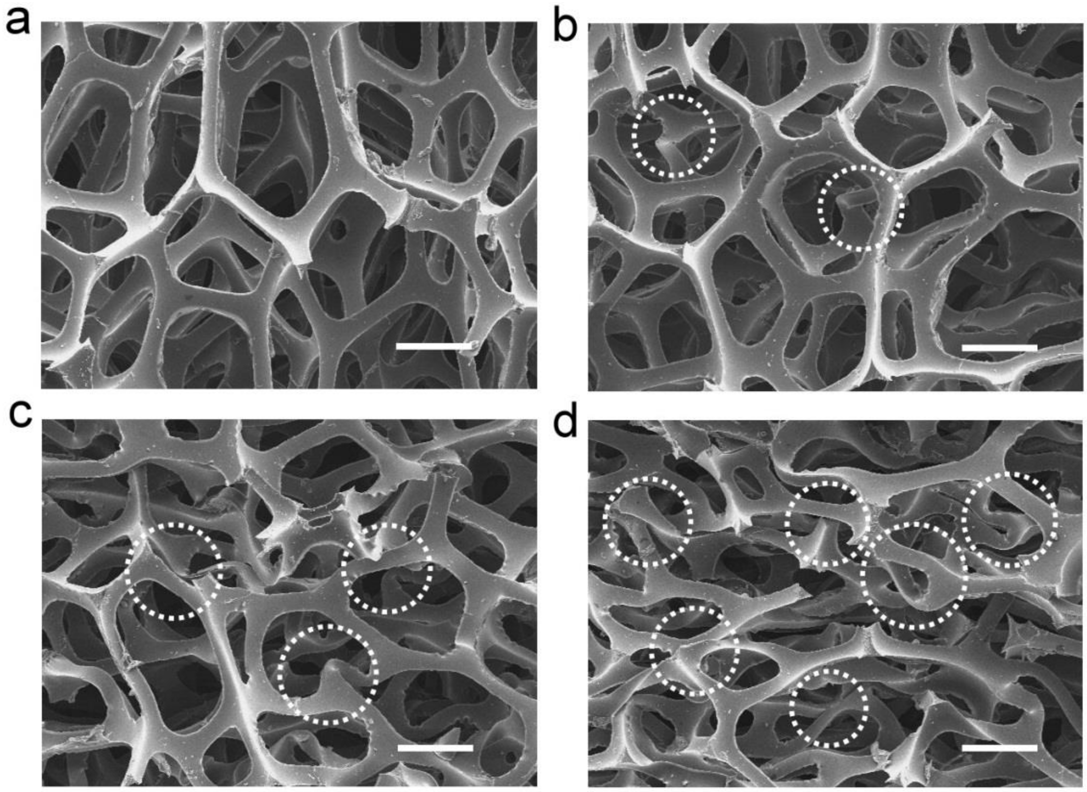

Supplementary Figure 8 | Microstructure characterizations of MFSOTE material with increasing compressive strain. SEM images of PU-PEDOT:PSS based MFSOTE material under different compressive strain of (**a**) 0%, (**b**) 10%, (**c**) 40% and (**d**) 60%, respectively. Scale bars: 200 μm.

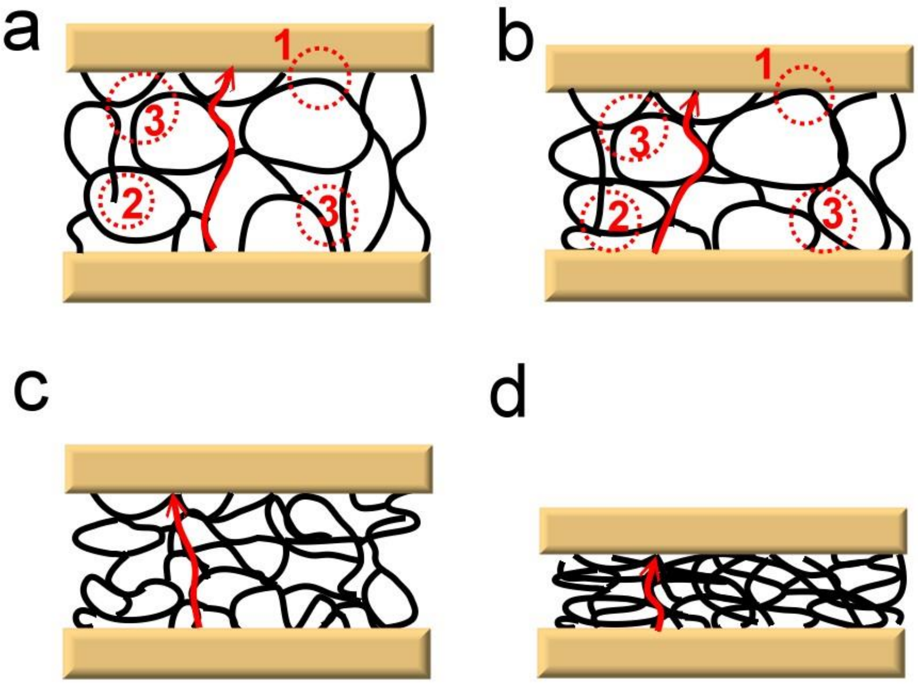

Supplementary Figure 9 | Schematic of pressure-sensing mechanism of MFSOTE-based devices with compressive strain of (a) 0%, (b) 10%, (c) 40%, and (d) 60 %. The red number of 1, 2, 3 in **(a)** and **(b)** indicate: ① the PU deformation induced contact change at electrode/active interface, ② the contact change induced by the fractured pore, and ③ the contact change resulting from the twisted cell. The red arrow displays the charge transport direction and length of the MFSOTE devices under different strains. Large strain leads to decreased contact resistance and internal resistance.

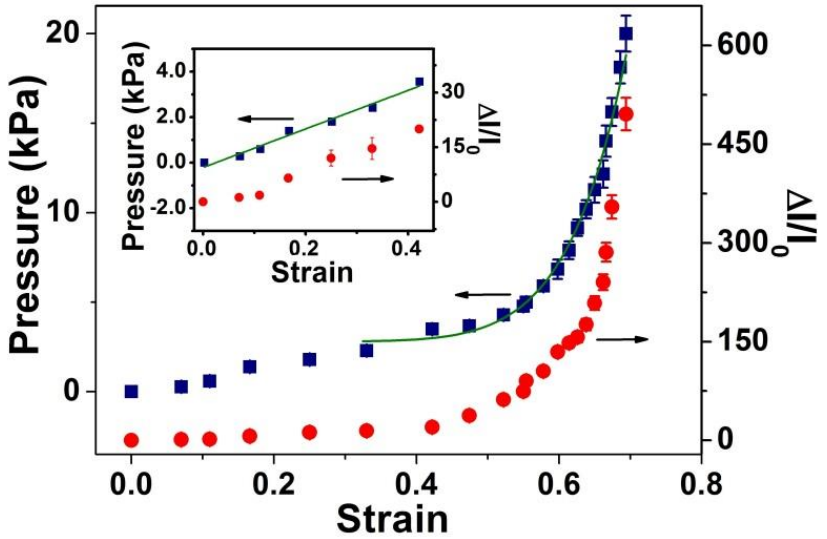

Supplementary Figure 10 | Mechanical and electrical measurements of a MFSOTE device with increasing compressive strain. Trade-off relationship between pressure and current change versus strain of the MFSOTE based devices. The error bars represent the standard deviation.

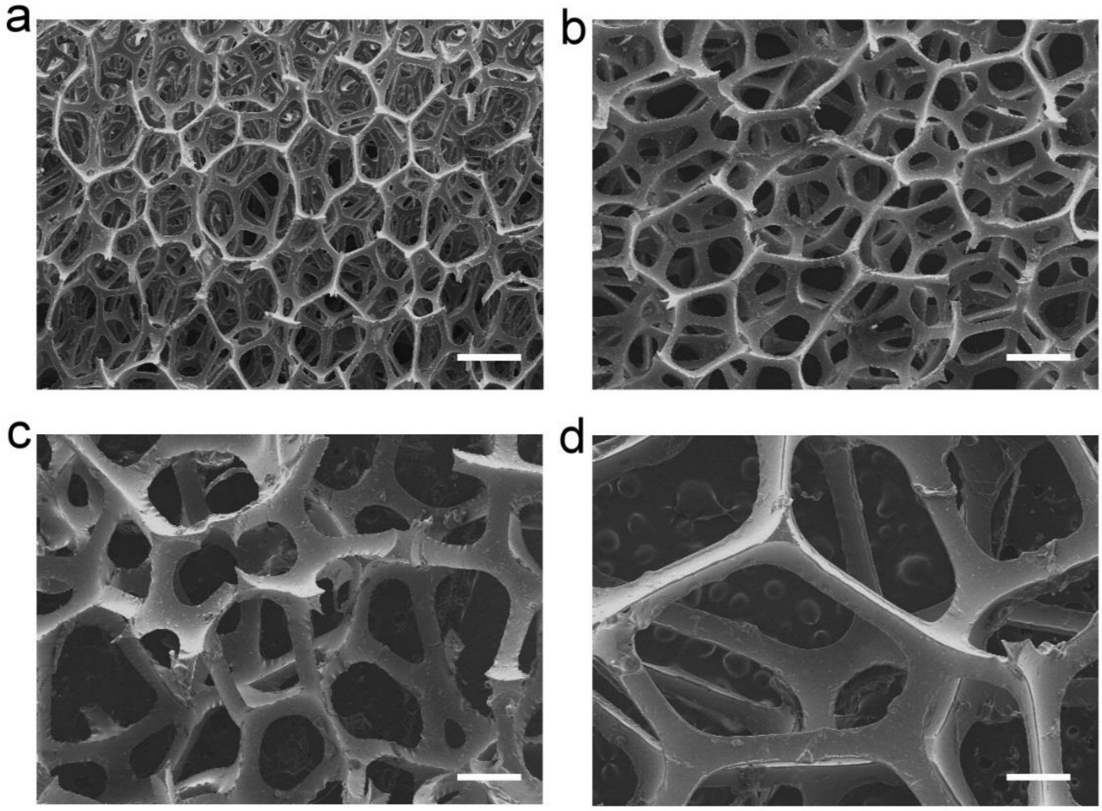

Supplementary Figure 11 | Microstructure characterizations of MFSOTE materials with different pore densities. SEM images of MFSOTE material with pore densities of (**a**) 60, (**b**) 40, (**c**) 35 and (**d**) 25 pores per inch (ppi), respectively. Scale bars: 500 μm.

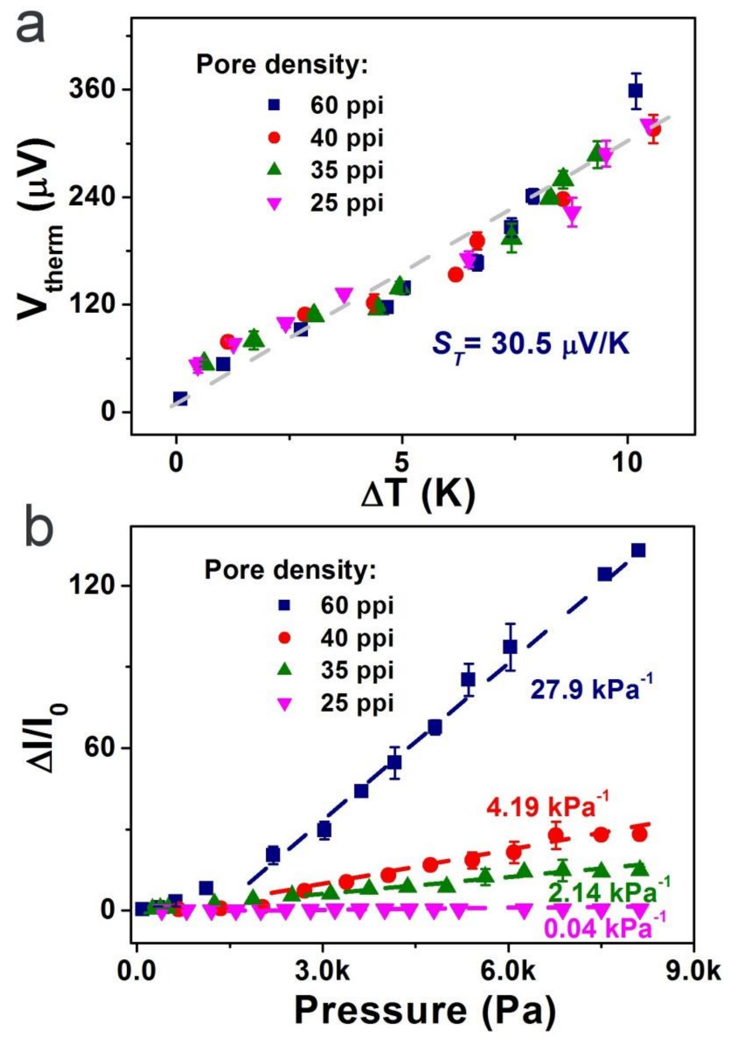

Supplementary Figure 12 | Comparison of sensing properties of MFSOTE devices with different pore densities. (**a**) Measured output voltage of PEDOT:PSS based MFSOTE devices with different pore densities as a function of temperature difference. (**b**) Measured current change of PEDOT:PSS based MFSOTE devices with different pore densities as a function of pressure. The error bars in the graph **a** and **b** were the standard deviation.

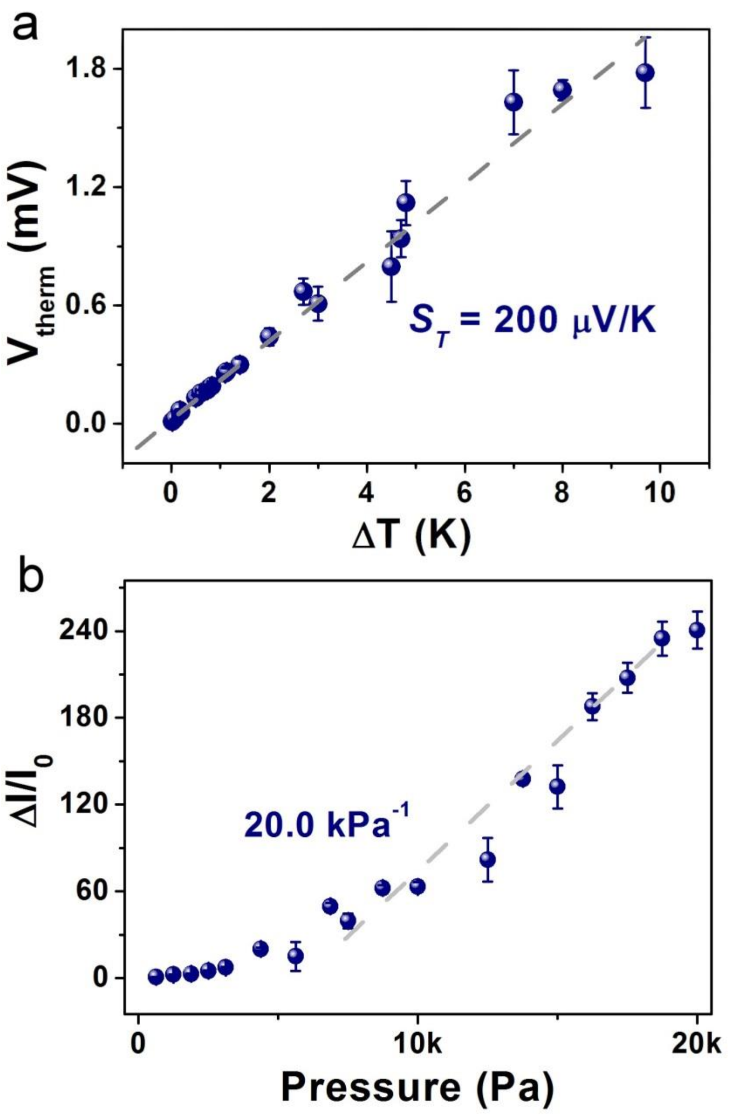

Supplementary Figure 13 | Sensing properties of PBTTT based MFSOTE devices. (**a**) Measured output voltage of PBTTT based MFSOTE devices as a function of temperature difference. The devices were pretreated with $NOPF_6$ to achieve higher conductivity. (**b**) Measured current response of PBTTT based MFSOTE devices as a function of pressure. The error bars in the graphs represent one standard deviation.

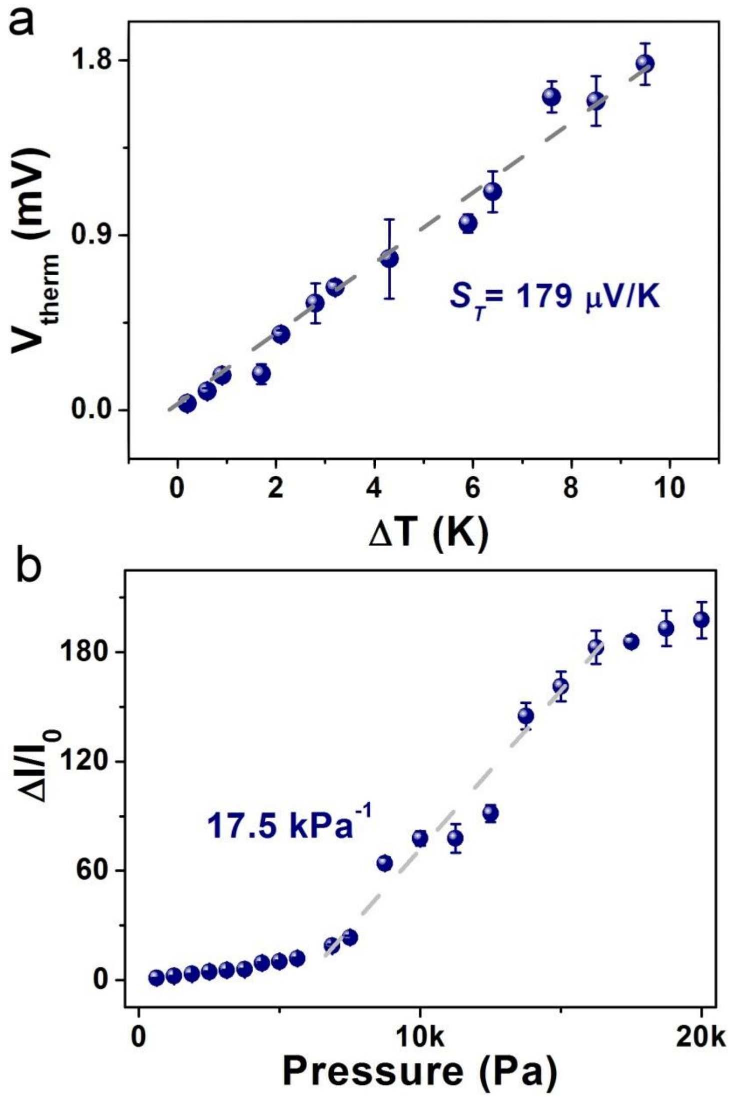

Supplementary Figure 14 | Sensing properties of P3HT based MFSOTE devices. (**a**) Measured output voltage of P3HT based MFSOTE devices as a function of temperature difference. The devices were pretreated with $NOPF_6$ to achieve higher conductivity. (**b**) Measured current response of P3HT based MFSOTE devices as a function of pressure. The error bars in the graphs represent the standard deviation.

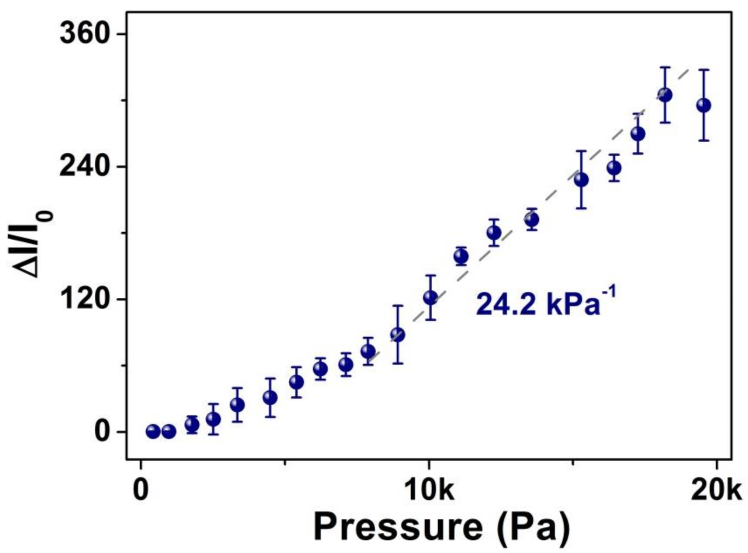

Supplementary Figure 15 | Pressure induced current response of PEDOT:PSS based MFSOTE devices. The PU frames were pre-treated with o-dichlorobenzene solvent and the error bars represent the standard deviation.

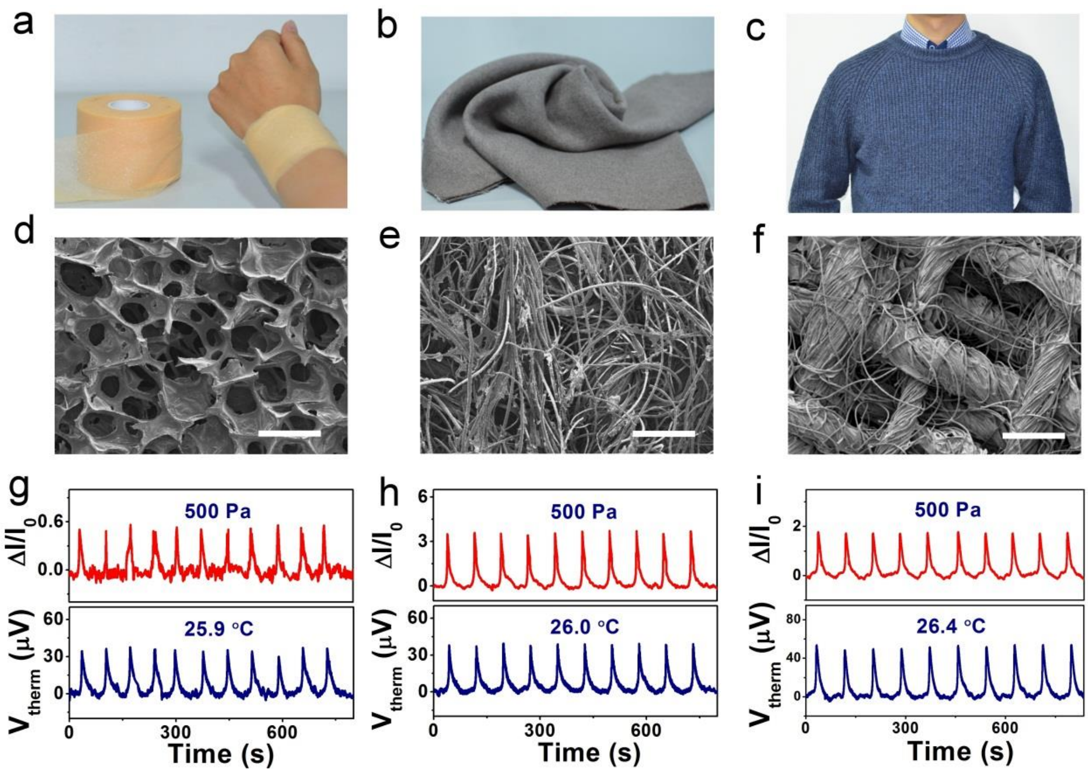

Supplementary Figure 16 | MFSOTE materials based on supporting-frame with different microstructured textures and their corresponding sensing characterizations. Photographs (**a-c**) and SEM images (**d-f**) of the supporting frame: (**a, d**) porous polyurethane (PU) based bandage, (**b, e**) intertwined cellulose and (**c**, **f**) grid-structured weaving cashmere. Scale bars: 500 μm. Graph **g-i** are the corresponding temperature and pressure sensing properties of (**g**) porous PU based bandage, (**h**) intertwined cellulose and (**i**) grid-structured weaving cashmere based MFSOTE sensors. The measured Seebeck coefficient are 29.5±5.0 μV/K, while the pressure sensing sensitivities of porous PU based bandage, intertwined cellulose and grid-structured weaving cashmere based MFSOTE sensors are 1.2 kPa^{-1}, 17.5 kPa^{-1} and 4.6 kPa^{-1}, respectively.

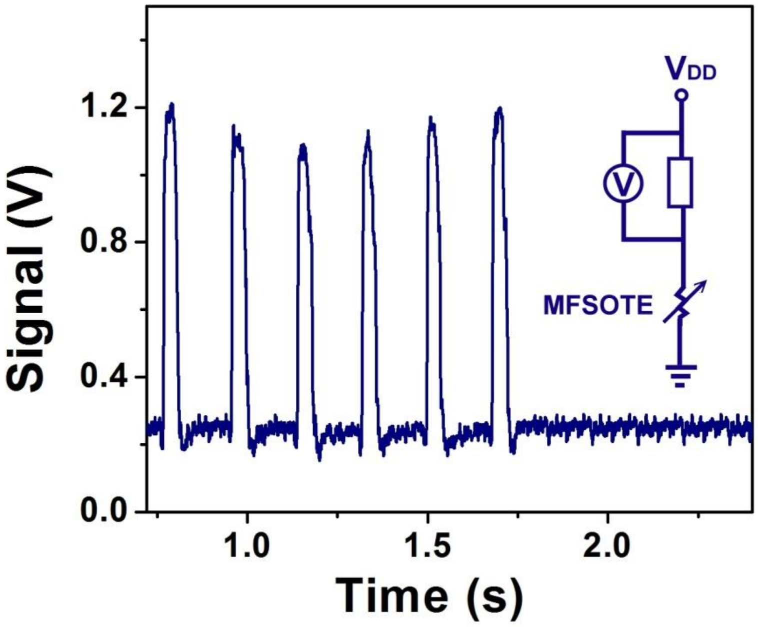

Supplementary Figure 17 | Instant pressure response of the MFSOTE based pressure sensor. Oscilloscope recorded time-resolved response of a MFSOTE device under loading-unloading pressure cycles. The inset shows the electrical equivalent circuit of the measurement system.

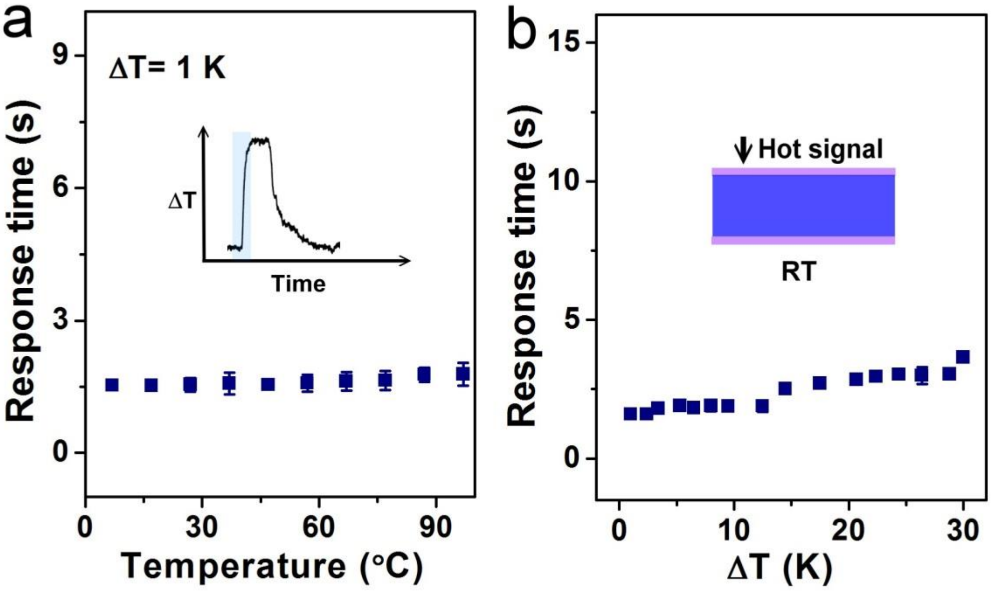

Supplementary Figure 18 | Temperature sensing response and relaxation time of MFSOTE device with different environmental temperatures and temperature differences. (**a**) Temperature sensing response time of MFSOTE devices with ΔT=1 K at various environmental temperature. (**b**) Response time as a function of ΔT. The error bars represent the standard deviation.

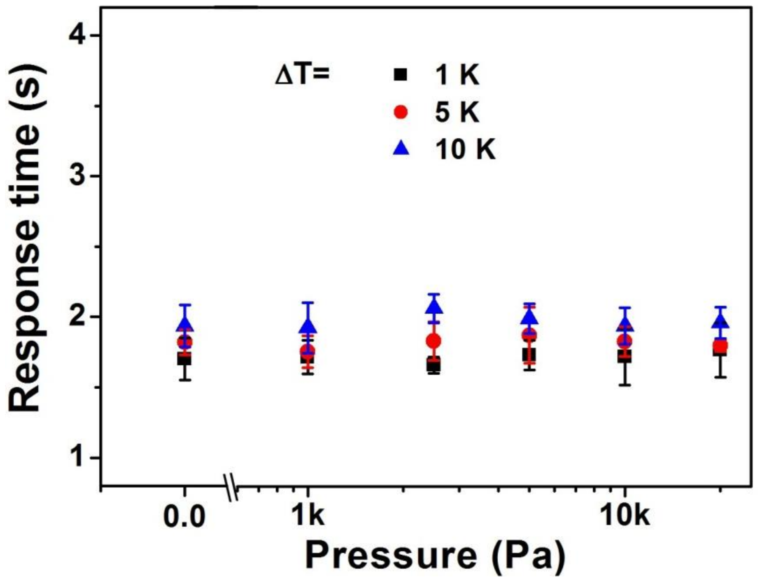

Supplementary Figure 19 | Temperature sensing response time of the MFSOTE device as a function of biased pressure. The error bars were the standard deviation.

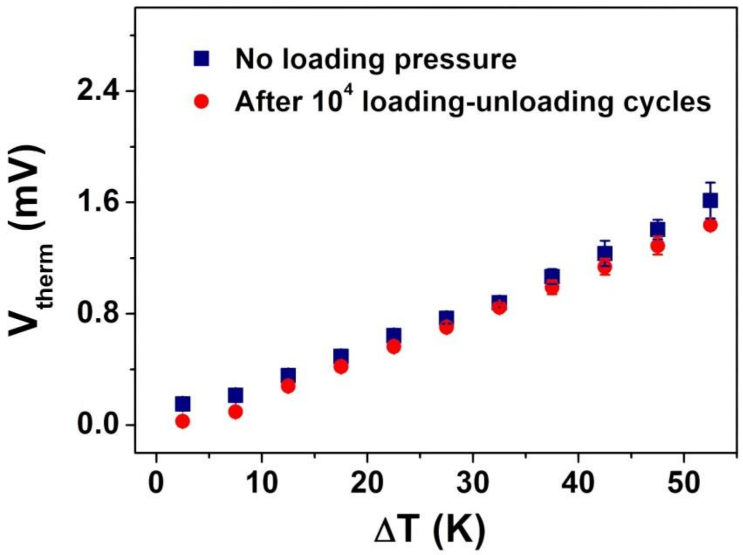

Supplementary Figure 20 | Stable temperature sensing performance after 10^4 loading-unloading pressure cycles. The error bars represent the standard deviation.

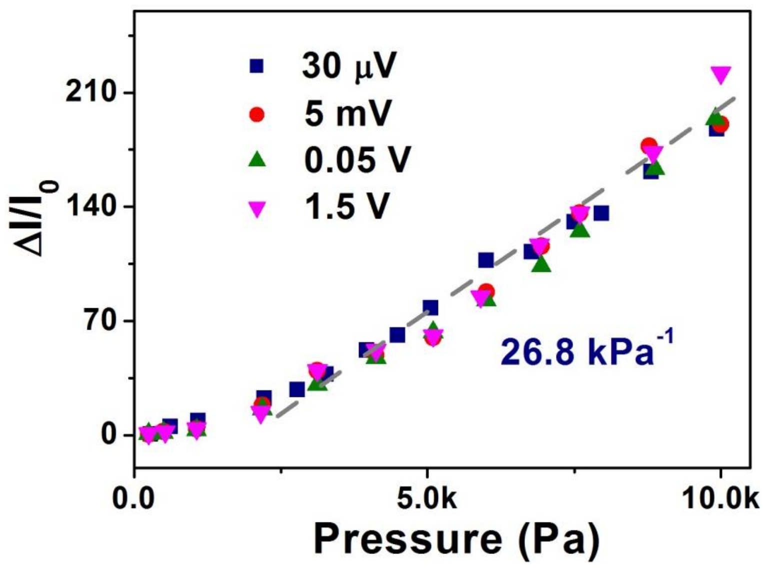

Supplementary Figure 21 | Pressure sensing performance of MFSOTE devices driven by biased voltage.

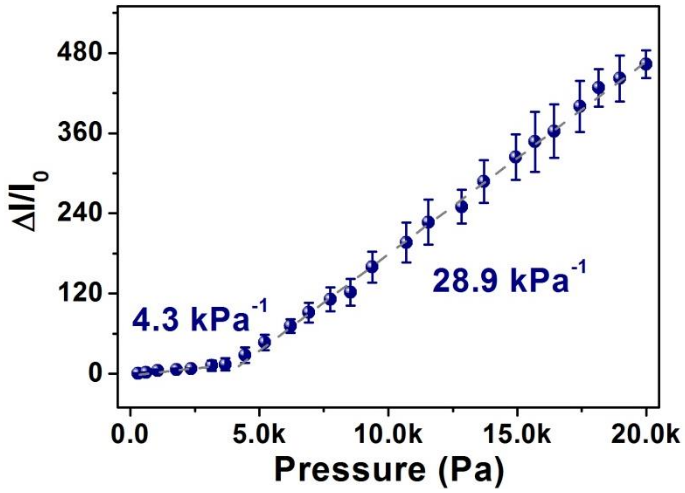

Supplementary Figure 22 | Pressure dependent current responses of a self-powered MFSOTE device summarized from Figure 3b.

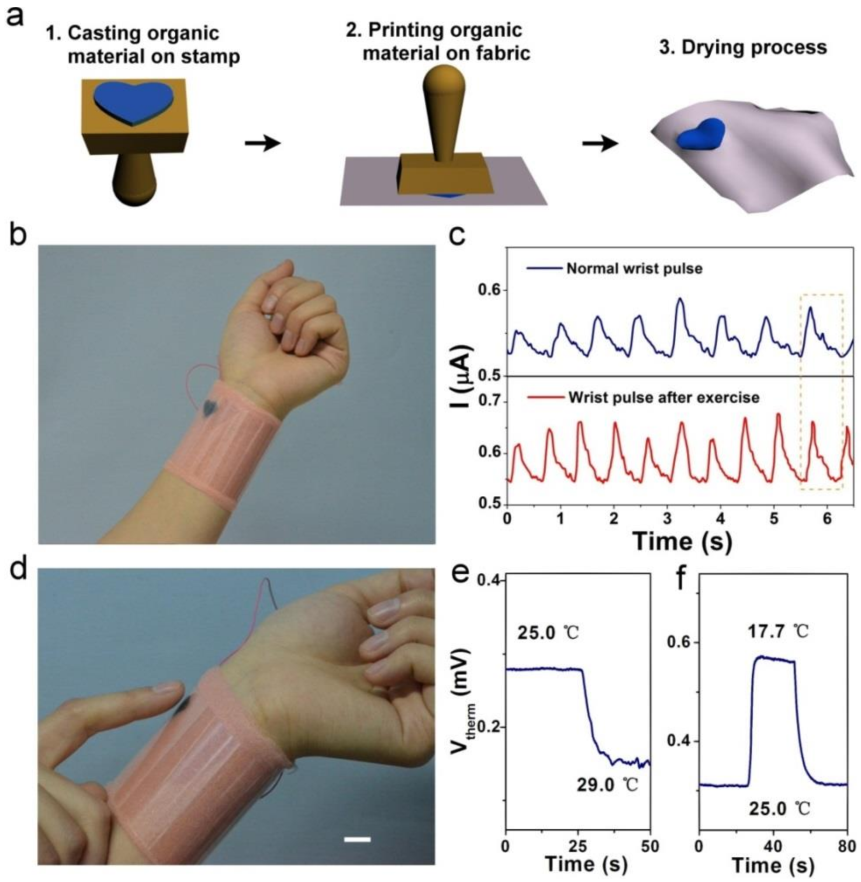

Supplementary Figure 23 | Fabrication and real-time monitoring performance of flexible MFSOTE devices. (**a**) Schematic process for the fabrication of a flexible sensor. (**b**) Photograph showing the sensor directly attached above the artery of the wrist. (**c**) The real-time monitoring of wrist pulse under normal and after physical exercise conditions, respectively. The calculated radial augmentation AI_r and ΔT_{DVP} are 0.54 and 175 ms, respectively, in a calm state, and the values are 0.57 and 139 ms, respectively, during exercise. The calculated parameters are characteristic values for a healthy adult female. (**d**) Photograph and (**e**) Output voltage response of a wearable device to a contact-free approaching of a finger. The distance between the finger and the device is 2 mm. Scale bar: 1 cm. (**f**) Output voltage response of a wearable MFSOTE device from a lab (25.0 ℃) to outdoors (17.7 ℃).

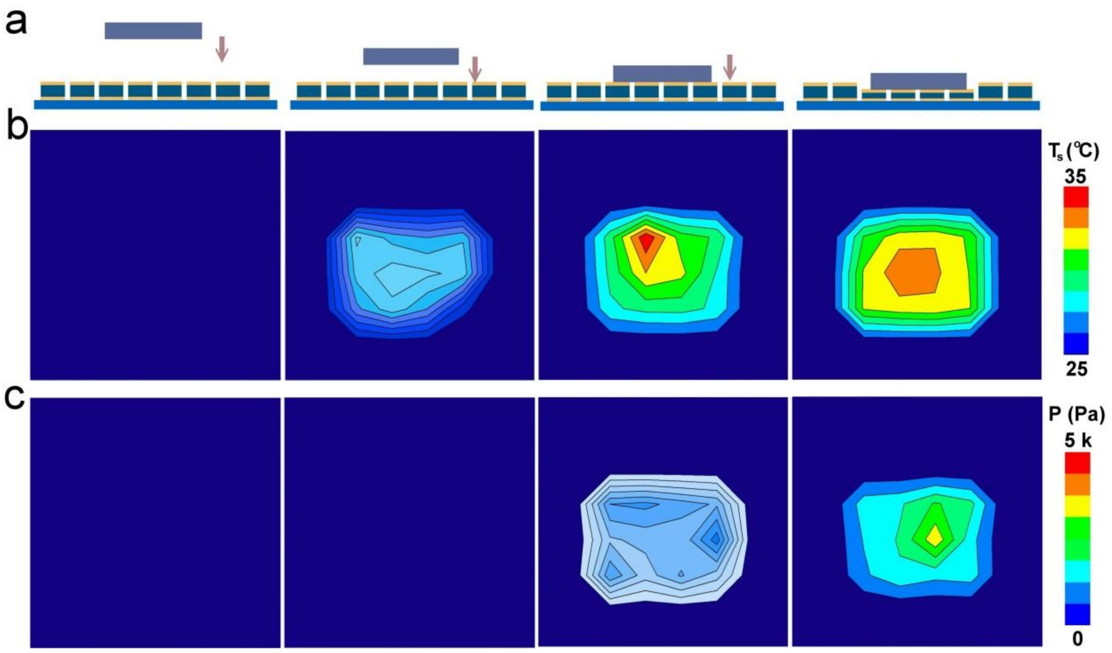

Supplementary Figure 24 | Real-time monitoring of temperature and pressure using a self-powered MFSOTE array. (**a**) Schematic of four locations of heating Peltier element relative to the MFSOTE sensor array. Graph **b** and **c** display the corresponding temperature and pressure mapping profile of the pixel signals, respectively.

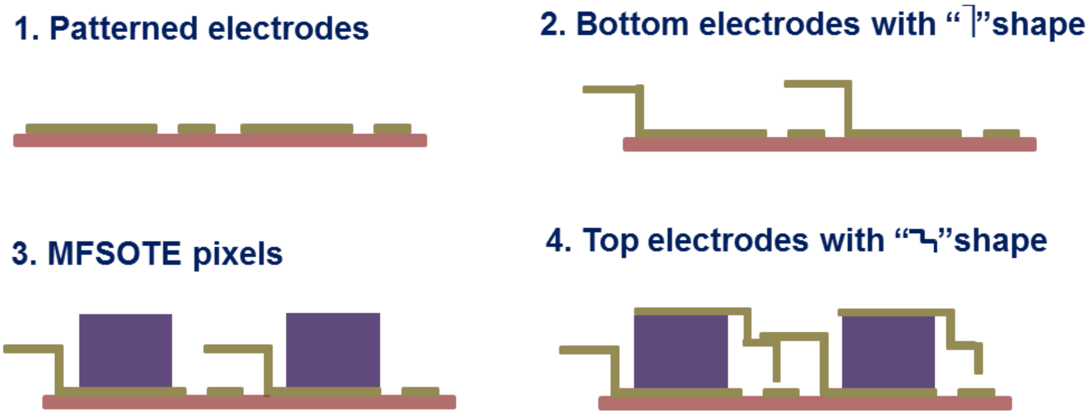

Supplementary Figure 25 | Schematic diagram of fabrication process of the multi-functional MFSOTE array.

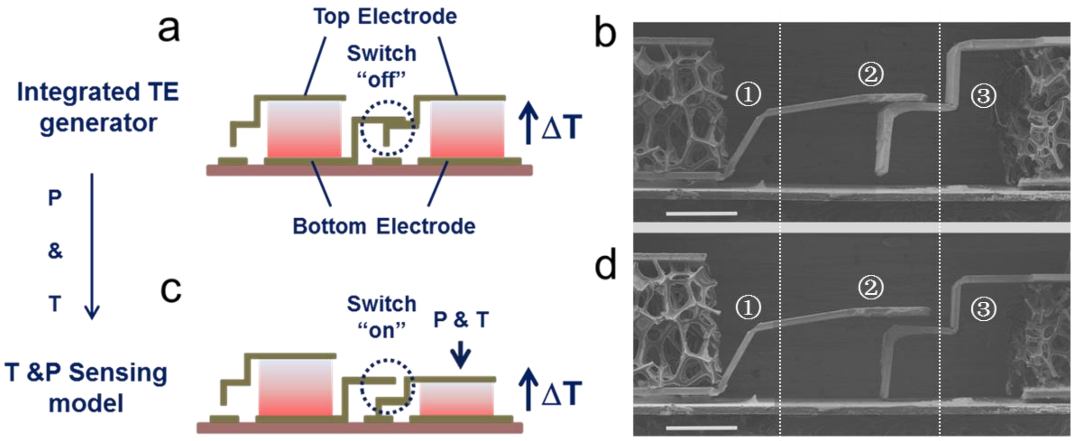

Supplementary Figure 26 | Schematic illustration of the switch of the sensing array. (**a**, **c**) Schematic illustration and (**b**, **d**) SEM images of the switch with (**a**, **b**) power generation and (**c**, **d**) stimuli sensing mode. The number of 1, 2, 3 in (**b**) and (**d**) indicate: ① the bottom electrode, ② the switch, and ③ the top electrode. Scale bars: 1 mm.

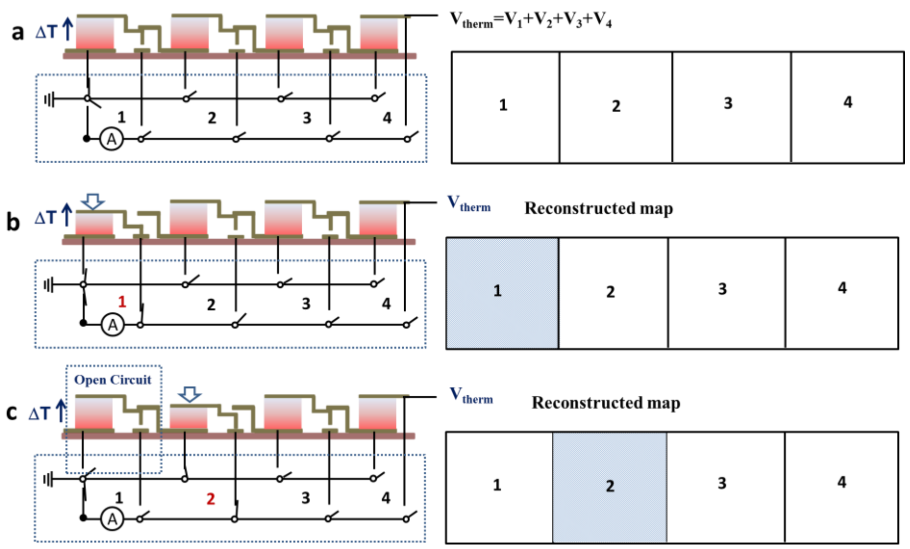

Supplementary Figure 27 | Schematic diagram of sensing array. (**a**) Circuit diagram of the device array under operation in the power generation model. Circuit diagrams of the device array when (**b**) device 1 or (**c**) device 2 is in contact with the additional object. The pixel addressing and reduction of the current leakage are achieved utilizing the matrix switch system.

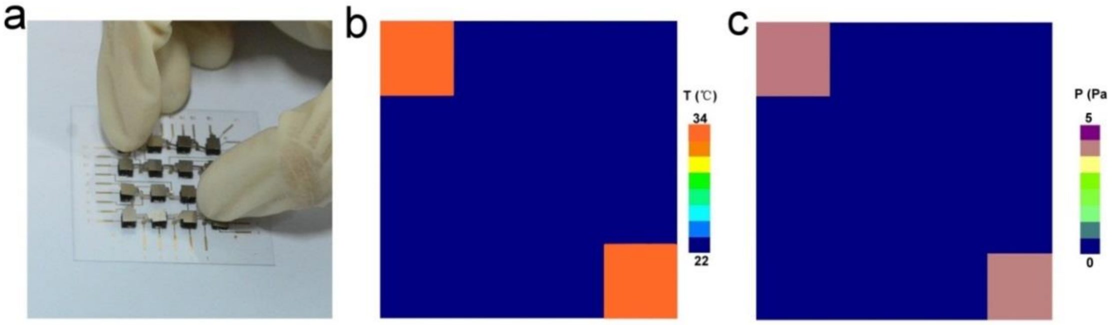

Supplementary Figure 28 | The performance of MFSOTE-based sensing array. (**a**) Photograph of integrated MFSOTE array with two pixels in contact with fingers. (**b**) Temperature and (**c**) Pressure mapping profile of the functional array.

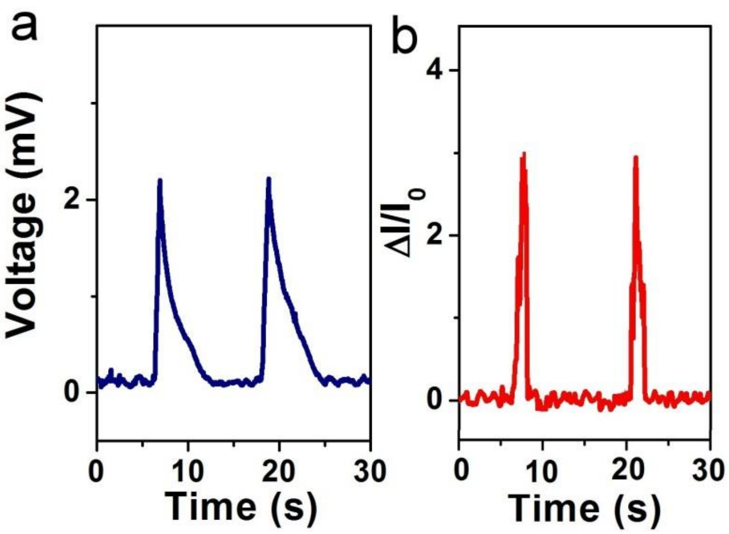

Supplementary Figure 29 | Real-time monitoring of the fabric glove based MFSOTE device. The measured (**a**) output voltage and (**b**) current response for a coupling signal when the hot-side temperature is 130℃.

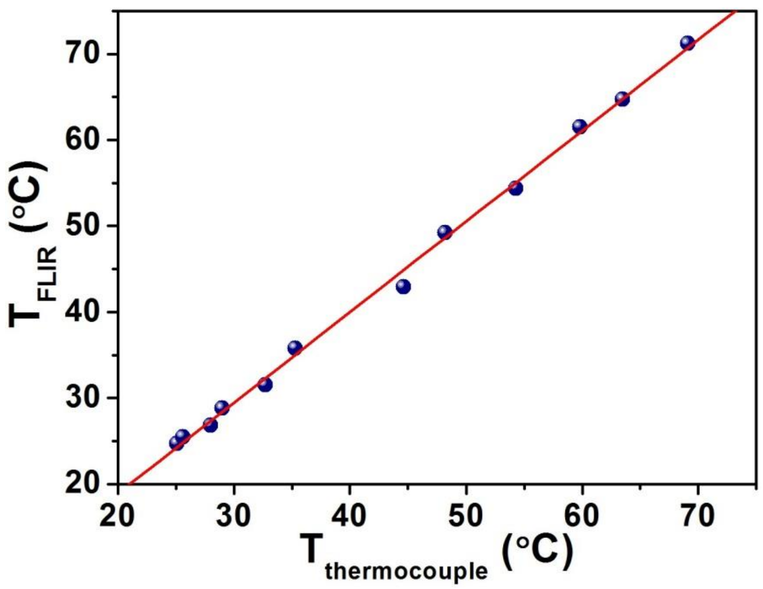

Supplementary Figure 30 | Calibration curve of the infra-red camera A300 and thermocouples.

Supplementary Notes

Supplementary Note 1. Temperature sensing mechanism

For the devices, the temperature response is based on the thermoelectric properties of the MFSOTE materials. The temperature of the stimuli (T_s) can be given by[1]:

$$T_s = T_0 + \Delta T \quad (1)$$

$$\Delta T = \frac{V_{therm}}{S_T} \quad (2)$$

where V_{therm} is the output voltage, S_T is the Seebeck coefficient, ΔT is the temperature difference and T_0 is the constant temperature of the environment.

Supplementary Note 2. Pressure sensing mechanism

The piezoresistive effect of the MFSOTE materials contributes to their pressure response. For a MFSOTE device, the pressure-induced current change is determined by the contact resistance of the electrode/MFSOTE, contact resistance of neighboring polyurethane (PU)-PEDOT:PSS pores and resistance changes result from the decreased thickness of the compressed devices (Supplementary Figure 8 and 9). All the resistance changes are dominated by the pressure induced strain with two regimes, as reflected by the fact that the piezoresistive responses of the device are comparable to the mechanical responses of MFSOTE materials (Supplementary Figure 10). Large strain on the device contributes to both reduced contact resistance and significantly decreased internal resistance.

For the open-cell foams, the mechanical properties can be divided into two operational regimes. In the low strain ($\varepsilon<0.4$) regime, the strain-pressure relationship

can be expressed as[2-4]

$$P = E\varepsilon \ (\varepsilon<0.4) \tag{3}$$

where P is the biased pressure which reveals the stress in the compressive process, E is the elastic modulus of the MFSOTE materials, ε is the compressive strain. In contrast, a high strain gives a different strain-pressure relationship, which can be given by[3,5]

$$P = K\varepsilon^{n_f} \ (\varepsilon>0.4) \tag{4}$$

where K is a parameter related to the Young's modulus of the solid cell wall material, n_f, which is affected by the density, is strain-hardening exponent of the foam obtained by fitting a power-law relation to the data points of the stress-strain curve. The strain-pressure relationship described above can be well verified by fitting the measured pressure-strain relationship of our MFSOTE devices since two strain-pressure relationship (P(kPa)=7.8ε and $P(kPa)=2.8+398\varepsilon^{8.8}$) were obtained in the low and high strain regime, respectively (Supplementary Figure 10).

As mentioned above, the current change is determined by the strain of the composite (PU-PEDOT:PSS) upon biased pressure. As two different pressure-strain relationships are obtained within two different regimes, different sensitivity $((\Delta I/I_0)/\Delta P)$ in the low- and high-pressure regime can be obtained.

Supplementary References

1. Rowe, D. M. *Thermoelectrics Handbook: Macro to Nano* (CRC, FL, 2006).

2. Gong, L., Kyriakides, S., & Jang, W. Y. Compressive response of open-cell

foams. Part I: Morphology and elastic properties. *Int. J. Solids. Struct*. **42**, 1355–1379 (2005).

3. Ashby, M. F. & Mehl R. E. The mechanical properties of cellular solids. Metall. *Mater. Trans. A* 14A, 1755–1769 (1983).
4. Gibson L. J. & Ashby M. F. Cellular solids: structure and properties (Pergamon, New York, 1988).
5. Amsterdam, E., de Vries, J. H. B., De Hosson J. Th. M. & Onck, P. R. The influence of strain-induced damage on the mechanical response of open-cell aluminum foam. *Acta Mater.* **56**, 609–618 (2008).
